# *Akkermansia muciniphila* reverses neuronal atrophy in *Negr1* knockout mice with depression-like phenotypes

**DOI:** 10.1080/19490976.2025.2508424

**Published:** 2025-05-19

**Authors:** Hee-Gon Hwang, Ji-Woo Park, Hyo-Jin Lee, Moon Yi Ko, Minhan Ka, Yun Kyung Lee, Jaeyoon Choi, Su-A In, Ye-Eun Lee, Soojin Lee, Min-Soo Kim, Jeong-Yoon Kim

**Affiliations:** a Department of Microbiology and Molecular Biology, Chungnam National University, Daejeon, Republic of Korea; b Department of Advanced Toxicology Research, Korea Institute of Toxicology, Daejeon, Republic of Korea; c Department of Integrated Biomedical Science, Soonchunhyang Institute of Medi-Bio Science, Soonchunhyang University, Cheonan, Republic of Korea

**Keywords:** *Akkermansia muciniphila*, genetic predisposition, microbiome, depression

## Abstract

Genetic predispositions can shape the gut microbiome, which in turn modulates host gene expression and impacts host physiology. The complex interplay between host genetics and the gut microbiome likely contributes to the development of neuropsychiatric disorders, yet the mechanisms behind these interactions remain largely unexplored. In this study, we investigated the gut microbiota in *Negr1* knockout (KO) mice, which exhibit anxiety- and depression-like behaviors, as NEGR1 (neuronal growth regulator 1) is a cell adhesion molecule linked to neuronal development and neuropsychiatric disorders. Our findings show significant early-life alterations in the gut microbiota composition of *Negr1* KO mice, most notably a marked reduction in *Akkermansia* spp. along with reduced dendritic arborization and spine density in the nucleus accumbens (NAc) and the dentate gyrus (DG) of the hippocampus. Remarkably, daily administration of an *Akkermansia* strain isolated from wild-type mice reversed the neuronal structural abnormalities and ameliorated anxiety- and depression-like behaviors in *Negr1* KO mice. Transcriptomic profiling revealed upregulation of mitochondrial genome-encoded genes in the NAc and hippocampus of *Negr1* KO mice, along with a predisposition toward a pro-inflammatory state in the colon of *Negr1* KO mice. The *Akkermansia* supplementation downregulated these mitochondrial genes in the NAc and hippocampus and upregulated genes involved in T cell activation and immune homeostasis in the colon. These findings demonstrate a novel gene–microbiome interaction in the pathophysiology of *Negr1* KO mice, positioning *Akkermansia* spp. as a key mediator that improves neuronal atrophy and modulates anxiety- and depression-like behaviors. Our study provides compelling evidence for bidirectional interactions between host genetics and the gut microbiome in modulating neuropsychiatric phenotypes, offering new insights for addressing genetically influenced mental disorders.

## Introduction

Major depressive disorder (MDD) is a prevalent mental disorder with a significant impact on individuals’ well-being and social functioning. The pathogenesis of MDD is highly complex, involving genetic, environmental, and psychological factors.^1^ Recent research has highlighted the potential role of the gut microbiome in developing MDD.^2^ Studies have indicated that individuals diagnosed with depression, as well as animal models exhibiting depression-like behaviors, have alterations in the gut microbiome, compared to normal subjects.^3–6^ This microbiome dysbiosis can be characterized by altered diversity and abundance of bacterial species.^4,7,8^ Notably, experiments involving the transplantation of fecal microbiota from individuals with depression into germ-free rodents have replicated depression-like behaviors in the recipient rodents.^9,10^ Despite these findings, the intricate dynamics underlying the gut-brain connection in MDD remain poorly understood. It is especially unclear whether host genetics drive changes in the gut microbiome that subsequently play a causative role in the onset of depression-like behaviors. Therefore, investigating the relationship between genetic variations and the gut microbiome in depression-like behaviors, particularly using animal models with known genetic predispositions to depression-like behaviors, could be instrumental in clarifying this complex interplay.

Neuronal morphological defects in brain regions, such as the nucleus accumbens (NAc) and the dentate gyrus (DG) of the hippocampus, are crucial in the pathophysiology of depression.^11^ The NAc is a core region of brain reward circuitry and a center for emotionally salient information,^12^ and neuronal atrophy in the NAc can lead to aversive behaviors, such as social avoidance and anhedonia.^13,14^ The DG is vital for cognition, sensory memory, and emotional processing,^15^ and decreased neurogenesis and neuronal atrophy in the DG have been linked to psychiatric disorders, including anxiety and depression^16,17^ and psychosocial stress, such as that associated with intimate partner violence.^18^ It has been reported that neuronal atrophy in the hippocampal CA3 region was observed in germ-free mice,^19^ suggesting that the gut microbiome may influence brain functions through regulating neuronal morphology. However, specific members of the gut microbiome that influence neuronal morphology and their mechanisms of action in neuronal structural remodeling are largely unknown.

*Negr1* is recognized as a risk gene for depression. For instance, *Negr1* is highly expressed in the brain, particularly in the cortex, hippocampus, and striatum.^20^ It plays an important role in brain development and neuronal migration, maintenance of dendrites, and adult neurogenesis.^21–24^ Genome-wide association studies reported that single nucleotide polymorphism of *Negr1* is highly associated with MDD^25–27^ and a transcriptome-wide association study also identified *Negr1* as one of the genes associated with depression.^28^ Additionally, the expression level of *Negr1* decreased in postmortem hippocampal tissue of patients with depression compared to those of healthy individuals.^29^ In animal studies, restraint stress decreased *Negr1* expression levels in the medial prefrontal cortex in rats,^30^ and NEGR1 deficiency has been linked to depression-like behaviors in mice.^24,31^ As such, *Negr1* knockout (KO) mice could be a suitable model for studying the interplay between genetic variations and the gut microbiome in the context of depression-like behaviors.

In this study, we investigated whether NEGR1 deficiency alters gut microbiota composition and whether these changes are linked to neuronal atrophy and anxiety- and depression-like behaviors in *Negr1* KO mice. *Akkermansia* spp. emerged as a key taxon strongly associated with these phenotypic abnormalities. Transcriptomic profiling identified molecular pathways through which *Akkermansia* may influence gut–brain interactions, including the regulation of mitochondrial gene expression in the NAc and hippocampus, as well as immune homeostasis in the colon. These findings provide insights into how host genetic factors shape gut microbiome composition and, in turn, impact neuronal function and behavior.

## Results

### Negr1 knockout mice exhibit defective dendritic arborization and reduced spine density in the nucleus accumbens and dentate gyrus

**Figure 1.**
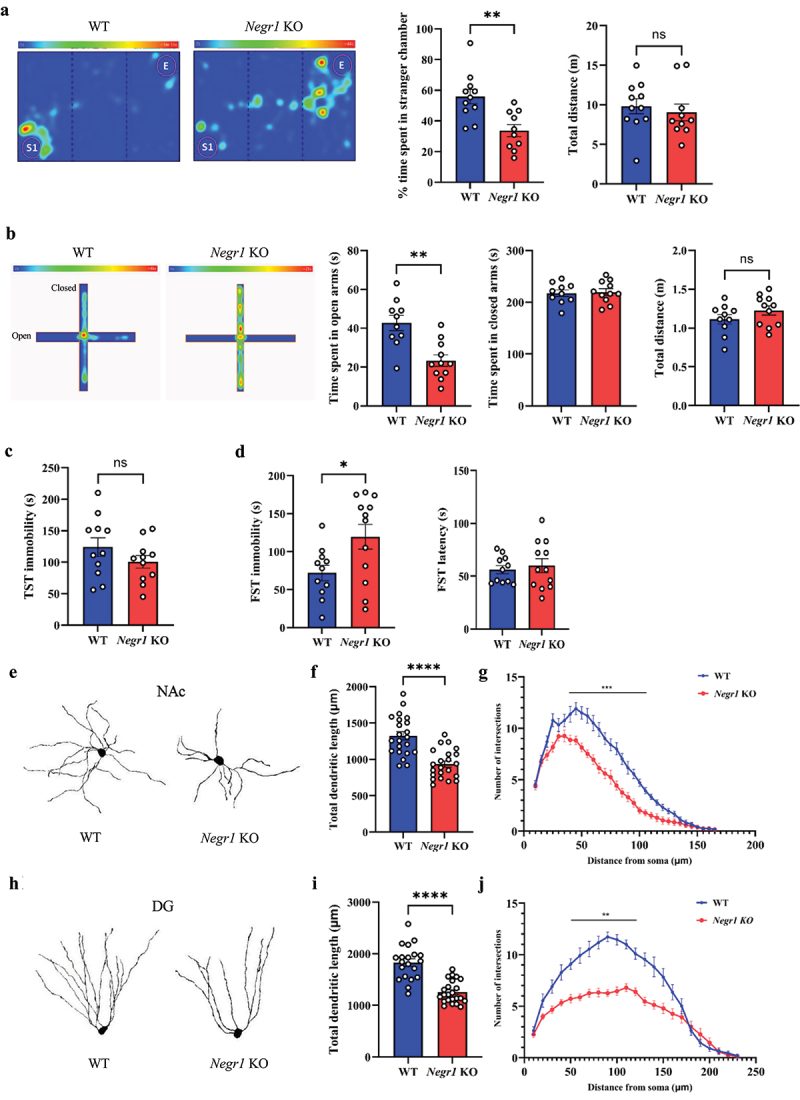
*Negr1* KO mice displayed depression-like behaviors and reduced dendritic arborization in NAc and DG neurons.

In this study, we investigated whether NEGR1 affects dendritic arborization of neurons in the NAc and DG, which are critically connected with depression phenotypes.^32,33^ We first conducted experiments to verify whether *Negr1* KO mice exhibit anxiety- and depression-like behaviors, as reported in previous studies.^20,24,31^ In the three-chamber sociability test, *Negr1* KO mice spent more time in the empty cage zone than in the stranger mouse cage zone, while WT littermate mice spent more time in the stranger cage zone (*p* < 0.05) (Figure 1(a)). In the elevated plus maze test, *Negr1* KO mice spent significantly less time in the open arm than WT mice (*p* < 0.05) (Figure 1(b)). Notably, the total distance moved in both tests did not differ significantly between groups, indicating that the observed behavioral differences are unlikely to be attributed to general locomotor deficits (Figure 1(a–b)). In the tail suspension test, the immobility time did not differ significantly between WT and *Negr1* KO mice (Figure 1c). However, in the forced swim test, *Negr1* KO mice exhibited significantly increased immobility time compared to WT mice (*p* < 0.05) (Figure 1(d)). These findings support the conclusion that NEGR1 deficiency contributes to anxiety- and depression-like behaviors in mice. These findings corroborate that NEGR1 deficiency leads to anxiety- and depression-like behaviors in mice. Subsequently, we examined neuronal morphology in the NAc and DG of *Negr1* KO mice. Neurons in these regions of *Negr1* KO mice showed shorter total dendritic length and reduced dendritic complexity compared to those of WT mice (*p* < 0.05) (Figure 1(e–j)). These morphological abnormalities in the neurons are likely associated with the observed anxiety- and depression-like behaviors in *Negr1* KO mice.

(a) Representative heatmaps and bar graphs of WT and *Negr1* KO mice for the three-chamber social test. Bar graph shows the time spent in zones with the stranger mouse (left) and the total distance moved during the test (right) (*n* = 11 for WT mice, *n* = 10 for *Negr1* KO mice). (b) Representative heatmaps and bar graphs of WT and *Negr1* KO mice for the elevated plus maze test. Bar graphs show time spent in the open (left) and closed (middle) arms and total distance moved (right) (*n* = 10 for WT mice, *n* = 11 for *Negr1* KO mice). (c) Bar graph of immobility time in the tail suspension test (TST) (*n* = 11 for WT mice, *n* = 11 for *Negr1* KO mice). (d) Bar graph of immobility time (left) and latency to first immobility (right) in the forced swimming test (FST) (*n* = 11 for WT mice, *n* = 12 for *Negr1* KO mice). (e) Representative reconstruction images of Golgi-stained medium spiny neurons in the NAc. (f, g) Quantitative analysis of NAc neuronal morphology (*n* = 22 neurons for both WT and *Negr1* KO mice from four brains). Total dendritic length (f). Dendritic complexity assessed by Sholl analysis (g). (h) Representative reconstruction images of Golgi-stained DG neurons. (i, j) Quantitative analysis of DG neuronal morphology (*n* = 20 for WT mice, *n* = 23 for *Negr1* KO mice from five brains). Total dendritic length (I). Dendritic complexity measured by Sholl analysis (j). *P* values were determined by (multiple) unpaired two-tailed t-test. *p < 0.05, **p < 0.01, ***p < 0.001, ****p < 0.0001. All data are presented as mean ± S.E.M.

### Community-level alterations in the gut microbiota of Negr1 knockout mice in early life

**Figure 2.**
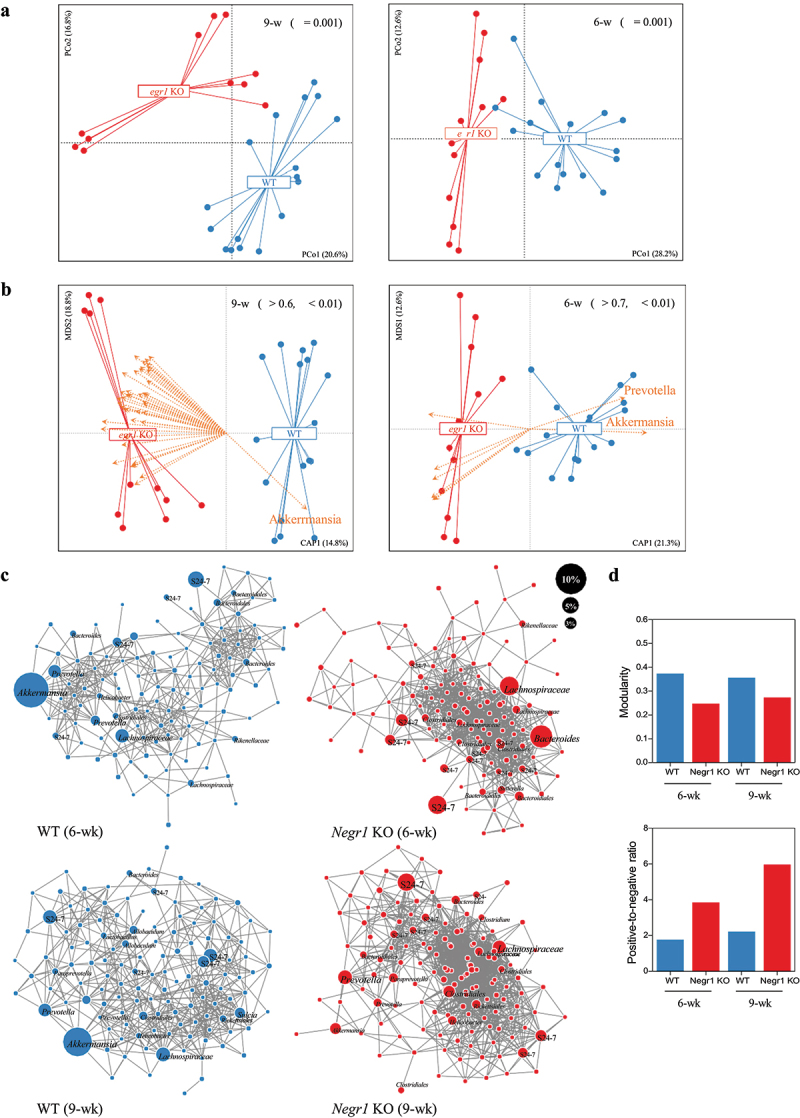
Alterations in the composition and network of the gut microbiota in *Negr1* KO mice.

The microbiome plays a vital role in the gut–brain axis underlying psychiatric disorders, including MDD.^34,35^ To investigate whether anxiety- and depression-like behaviors of *Negr1* KO mice are associated with alterations in the gut microbiota, we compared fecal microbiota between *Negr1* KO mice at the age of 9–11 wk (9 wk, *n* = 17) and age- and gender-matched WT mice (*n* = 12). To minimize potential bias arising from different birth dates and breeding cages, we collected fecal samples from mice representing at least three different birth dates and five different breeding cages. Amplicon sequencing of the 16S rRNA gene was conducted, and the fecal microbiota were compared based on ASVs, which represent biological variants in a sample prior to amplification and sequencing steps.^36^ The composition of fecal microbiota (442 ASVs, 222 ± 58 ASVs per sample) was compared using PCoA with Bray-Curtis dissimilarity for ASV abundance and Jaccard distance for ASV occurrence. The fecal microbiota differed significantly between *Negr1* KO and WT mice in both the abundance and occurrence of bacterial ASVs (*p* < 0.001) (Figure 2(a), Supplementary Fig. S1A), indicating that *Negr1* KO mice have an altered gut microbiota. To determine whether this alteration is initiated early in life, we compared the fecal microbiota composition between *Negr1* KO and WT mice at 6–7 wk of age (6 wk). The fecal microbiota comprised 411 ASVs (201 ± 52 ASVs per sample) and was compared based on the two distance matrices. Again, significant differences were found in both the abundance and occurrence of bacterial ASVs between Negr1 KO and WT mice (*p* < 0.001) (Figure 2(a), Supplementary Fig. S1A). These findings suggest that the altered microbiota of *Negr1* KO mice may be assembled in the intestine early in life.

(a) PCoA based on Bray-Curtis dissimilarity showing bacterial community composition at 9 wk (left) and 6 wk (right). Statistical significance was evaluated using Adonis with 999 permutations. (b) Discriminant ASVs between WT and *Negr1* KO mice at 9 wk (left) and 6 wk (right), predicted by correlation with the constrained axis of db-RDA for genotype based on Bray-Curtis dissimilarity. Statistical significance was evaluated using envfit with 999 permutations. (c) Co-occurrence networks for ASVs with >0.1% average abundance in each group, displaying positive correlations among ASVs in *Negr1* WT and KO mice at 6 wk (left panel) and 9 wk (right panel). Node size proportionally corresponds to the average relative abundance of ASVs within each group. Taxonomy was presented at nodes with >1% of average abundance. (d) Comparison of network modularity (upper panel) and the ratio of negative-to-positive associations (lower panel) between WT and *Negr1* KO mice.

We further investigated whether the alterations in the gut microbiota of *Negr1* KO mice could be attributed to differences in the biodiversity of gut microbiota. At both ages, *Negr1* KO mice exhibited a higher number of bacterial ASVs, indicative of species richness, compared to WT mice (*p* < 0.05) (Supplementary Fig. S1B). Pielou’s evenness, a measure of how evenly species are distributed within a community, was also significantly higher in *Negr1* KO mice than in WT mice (*p* < 0.05), though the significance weakened at the later age (Supplementary Fig. S1B). Accordingly, Shannon index, which measures community biodiversity, was higher in *Negr1* KO mice than in WT mice (Supplementary Fig. S1B). Taken together, alterations in species richness and abundance of gut microbiota in *Negr1* KO mice are associated with NEGR1 deficiency, which may contribute to anxiety- and depression-like behaviors alongside neuronal structural abnormalities.

### Low abundance of Akkermansia spp. correlates with community-wide alterations in the gut microbiota of Negr1 knockout mice

The majority of bacterial ASVs at both ages were assigned to four phyla (>1% average abundance). The *Bacteroidetes* (44.1 ± 14.5% in 6 wk; 37.9 ± 11.4% in 9 wk, average ± s.d.) and *Firmicutes* (37.9 ± 16.6% in 6 wk; 40.2 ± 16.2% in 9 wk) phyla were predominant, with a minor abundance of *Verrucomicrobia* (8.9 ± 10.2% in 6 wk; 8.6 ± 8.0% in 9 wk) and *Proteobacteria* (3.2 ± 2.2% in 6 wk; 4.3 ± 5.6% in 9 wk) phyla (Supplementary Fig. S1C). The four phyla encompassed 48 bacterial genera, of which nine genera corresponded to the most abundant taxa in the gut microbiota of *Negr1* mice at both ages (>1.0% average abundance): S24–7 (24.5 ± 10.8% in 6 wk; 21.4 ± 8.2% in 9 wk), *Akkermansia* (8.9 ± 10.2% in 6 wk; 8.6 ± 8.0% in 9 wk), *Prevotella* (6.0 ± 5.5% in 6 wk; 6.1 ± 4.0% in 9 wk), *Bacteroides* (5.0 ± 6.0% in 6 wk; 2.3 ± 2.1% in 9 wk), *Oscillospira* (3.2 ± 1.8% in 6 wk; 2.6 ± 1.7% in 9 wk), *Ruminococcus* of Lachnospiraceae (1.8 ± 1.9% in 6 wk; 1.9 ± 1.5% in 9 wk), *Lactobacillus* (1.4 ± 1.4% in 6 wk; 2.8 ± 3.5% in 9 wk), *Paraprevotella* (1.0 ± 1.8% in 6 wk; 2.3 ± 2.7% in 9 wk), and *Coprococcus* (0.9 ± 0.9% in 6 wk; 1.5 ± 1.2% in 9 wk) (Supplementary Fig. S1C). These core genera have been widely reported as predominant members in any previous studies of murine gut microbiota.^37,38^

To identify influential taxa driving community-wide alterations in the gut microbiota of *Negr1* KO mice, we examined correlations between the relative abundance of bacterial ASVs and an axis of db-RDA constrained to genotype with permutations. Significant correlations were found for eight ASVs (*r* > 0.7, *p* < 0.01) in the 6-wk *Negr1* KO mice and 43 ASVs (*r* > 0.6, *p* < 0.01) in the 9-wk *Negr1* KO mice with the constrained axes of db-RDA plots using Bray-Curtis dissimilarity (Figure 2(b)). Most of the significant ASVs were specific to *Negr1* KO mice, which corresponds to the high diversity of the microbiota in *Negr1* KO mice. Only one ASV had a significant correlation specific to WT mice at both ages (*r* = 0.8529 in 6 wk and *r* = 0.6262 in 9 wk), which was taxonomically assigned to the genus *Akkermansia*. A similar result was obtained from discriminant analysis using LEfSe.^39^ In total, 44 and 36 differentially abundant ASVs were identified in 6-wk and 9-wk *Negr1* mice, respectively (LDA score > 3.0, *p* < 0.01). The majority of the differentially abundant ASVs were enriched in *Negr1* KO mice, while the *Akkermansia* ASVs were enriched in WT mice at both ages (Supplementary Fig. S1D). Indeed, the relative abundance of the *Akkermansia* ASVs was significantly higher in WT mice than *Negr1* KO mice at both ages (*p* < 0.01) (Supplementary Fig. S1E). *Akkermansia* is a genus of Gram-negative, mucin-degrading bacteria belonging to the phylum *Verrucomicrobia*, known for its role in maintaining intestinal barrier integrity and regulating host metabolism and immune function.^38,40–42^ Given its crucial role in gut homeostasis and host–microbiome interactions, the limited development of *Akkermansia* spp. in early life may be associated with neuronal structural defects and depression-like behaviors caused by NEGR1 deficiency.

To understand microbial interactions of the altered gut microbiota observed in *Negr1* KO mice, we constructed co-occurrence networks by predicting positive and negative correlations among the bacterial ASVs (*r* > 0.4, *p* < 0.01). Microbial networks contained 741 and 1,288 correlations connecting 120 and 126 ASVs in WT and *Negr1* KO mice at 6 wk, respectively (Figure 2(c)). The number of microbial associations increased to 870 and 1,849 correlations connecting 127 and 140 ASVs at 9 wk in WT and *Negr1* KO mice, respectively (Figure 2(c)), indicating that microbial interactions are enriched along with host development. The microbial association network of WT mice at 6 wk consisted largely of three discrete groups: *Akkermansia-Prevotella*, *Lachnospiraceae-Clostridiales*, and *Bacteroidales*-S24-7 (recently referred to as *Muribaculaceae*) groups, which were sustained well at 9 wk (Figure 2(c)). However, the microbial associations of *Negr1* KO mice at 6 wk were constructed only with the *Bacteroides-Lachnospiraceae*-S24–7 group, and this single group was concentrated more at 9 wk (Figure 2(c)). This was evident in the microbial network properties: the modularity level was lower in *Negr1* KO mice than in WT mice, and the network of *Negr1* KO mice was dominated by positive correlations (Figure 2(d)). Environmental stress has been shown to destabilize microbial networks, making them less modular and dominated by positive association.^43^ This pattern has been observed in the gut microbiomes of human, rodent, and zebrafish under various perturbations.^44–47^ Accordingly, the gut microbiota of *Negr1* KO mice, characterized by low modularity and a high ratio of positive-to-negative associations, may reflect a state of stress induced by NEGR1 deficiency, and the altered microbial networks could be associated with the low development of *Akkermansia* spp.

### Akkermansia spp. ameliorates anxiety- and depression-like behaviors and restores neuronal structural abnormalities in Negr1 knockout mice

The observation of the low abundance of *Akkermansia* spp. in *Negr1* KO mice allowed us to hypothesize that *Akkermansia* spp. may contribute to the development of anxiety- and depression-like behaviors of *Negr1* KO mice. We attempted to obtain the cultural strains of *Akkermansia* spp. that have been locally evolved and adapted to specific conditions of WT mice. Ten strains of *Akkermansia* spp. were isolated from fecal samples of WT mice. The 16S rRNA gene sequences exhibited similarities greater than 99.5% among the isolates, and >99.5% and <97.0% with the 16S rRNA gene sequences of *A*. *muciniphila* Muc^T^ and *A*. *glycaniphila* Pyt^T^, respectively (Supplementary Fig. S2A). The same result was drawn from the sequence similarities of the V3-V4 regions of the 16S rRNA gene (Supplementary Fig. S2B). The phylogenetic analysis showed that the isolates positioned together with the *Akkermansia* ASVs in a single branch (Supplementary Fig. S2C), indicating that the *Akkermansia* cultural isolates were representative of the *Akkermansia* spp. specific to WT mice. The genotypes of the *Akkermansia* isolates and *A. muciniphila* Muc^T^ were further compared using ERIC-PCR.^48^ No differences were observed in the genomic fingerprints among the isolates, whereas distinct bands were evident in the fingerprints when comparing the isolates to *A*. *muciniphila* Muc^T^ (Supplementary Fig. S2D).

**Figure 3.**
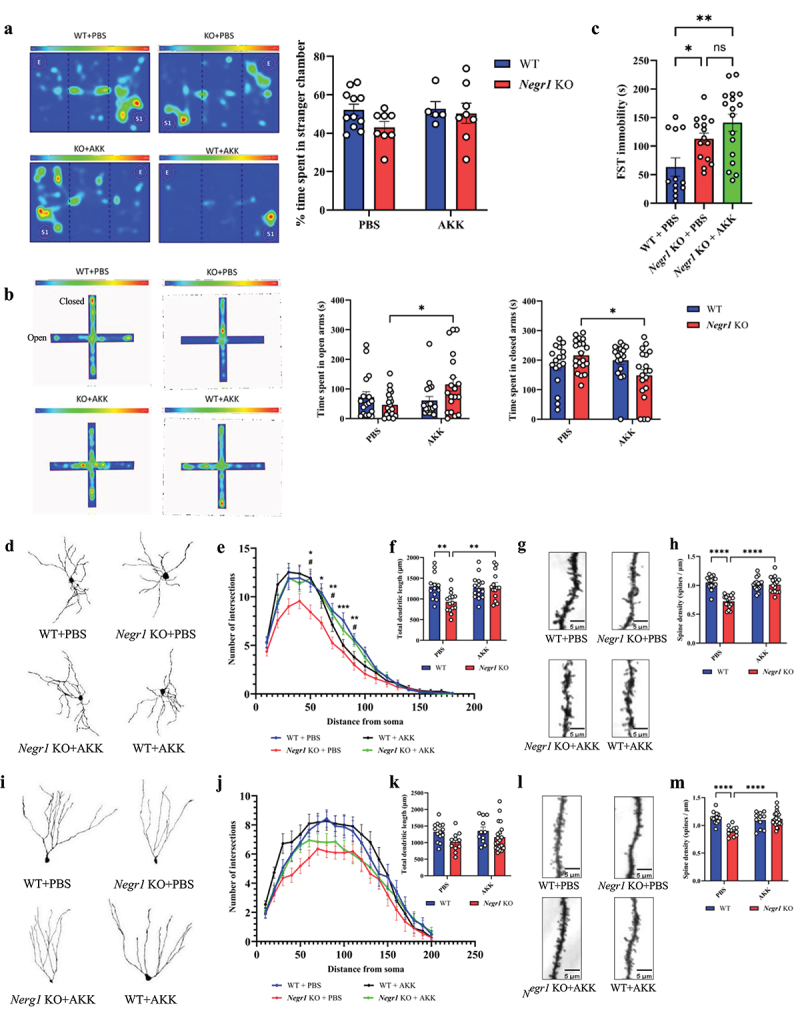
Administration of *Akkermansia* spp. ameliorates depression-like behaviors and restores dendritic arborization in *Negr1* KO mice.

To investigate whether the reduced presence of *Akkermansia* spp. is associated with anxiety- and depression-like behaviors and abnormalities in neuronal cell morphology in *Negr1* KO mice, we administrated a single strain (m3-2) of the *Akkermansia* isolates or PBS to *Negr1* KO and WT mice every weekday for 4 wk (Supplementary Fig. S3A). We then compared anxiety- and depression-like behaviors across four groups: WT+PBS, KO+PBS, KO+AKK, and WT+AKK. In the three-chamber sociability test, KO+AKK mice appeared to prefer the stranger mouse cage, whereas KO+PBS mice exhibited social deficits by favoring the empty cage zone, although the differences were not statistically significant (Figure 3(a)). In the elevated plus maze test, which measures anxiety-like behavior, KO+AKK mice spent more time in the open arms, indicating reduced anxiety, while KO+PBS mice spent less time in the open arms (Figure 3(b)). The total distance moved was comparable between the groups in both tests, indicating that general locomotor deficits did not contribute to the observed behavioral differences (Supplementary Fig. S4A-B). However, unlike the effects seen in the sociability and elevated plus maze tests, KO+AKK and KO+PBS mice showed no significant differences in the forced swim test and tail suspension test (Figure 3(c), Supplementary Fig. S4C-4D). These results suggest that *Akkermansia* treatment alleviates anxiety- and depression-like behaviors in *Negr1* KO mice, although its effects were not consistent across all behavioral assessments.

(a) Representative heatmaps and bar graphs from the three-chamber social test for each mouse group. Bar graph shows time spent in zones with and without the stranger mouse (*n* = 11 for WT+PBS, *n* = 8 for KO+PBS, *n* = 8 for KO+AKK, and *n* = 8 for WT+AKK). (b) Representative heatmaps and bar graphs from the elevated plus maze test. Bar graphs show time spent in open (left panel) and closed (right panel) arms (*n* = 17 for WT+PBS mice, *n* = 20 for KO+PBS mice, *n* = 19 for KO+AKK mice, and *n* = 18 for WT+AKK mice). (c) Bar graph of immobility time in the forced swimming test (*n* = 12 for WT+PBS mice, *n* = 15 for KO+PBS mice, and *n* = 17 for KO+AKK mice). (d) Representative neuronal reconstruction images of Golgi-stained medium spiny neurons in the NAc for each group. (e, f) Quantitative analysis of NAc neuronal morphology (*n* = 14 neurons for WT+PBS mice, *n* = 16 neurons for KO+PBS mice, *n* = 16 neurons for KO+AKK mice, and *n* = 15 neurons for WT+AKK mice, from three brains). Dendritic complexity measured by Sholl analysis (e). Quantification of total dendritic length (f). (g, h) Quantitative analysis of spine density in NAc neurons for each group (*n* = 14 neurons for WT+PBS mice, *n* = 16 neurons for KO+PBS mice, *n* = 16 neurons for KO+AKK mice, and *n* = 15 neurons for WT+AKK mice, from three brains). Representative images of Golgi-stained dendritic spines in NAc in each group (g). Quantification of dendritic spine density (h). (i) Representative neuronal reconstruction images of Golgi-stained DG neurons in each group. (j, k) Quantitative analysis of DG neuronal morphology (*n* = 14 neurons for WT+PBS, *n* = 11 neurons for KO+PBS, *n* = 22 neurons for KO+AKK, and *n* = 11 neurons for WT+AKK, from three brains). Dendritic complexity measured by Sholl analysis (j). Quantification of total dendritic length (k). (l, m) Quantitative analysis of spine density in NAc neurons for each group (*n* = 14 neurons for WT+PBS, *n* = 11 neurons for KO+PBS, *n* = 22 neurons for KO+AKK, and *n* = 11 neurons for WT+AKK, from three brains). Representative images of Golgi-stained dendritic spines in the DG (l). Quantification of dendritic spine density in the DG (m). *P* values were determined by one-way ANOVA with Tukey’s multiple comparison test or two-way ANOVA with Bonferroni’s multiple comparison test. *p or #*p* < 0.05, **p < 0.01, ***p < 0.001, ****p < 0.0001. *: WT+PBS vs. *Negr1* KO+PBS, #: *Negr1* KO+PBS vs. *Negr1* KO+AKK. All data are presented as mean ± S.E.M.

Next, we examined the effect of *Akkermansia* on neuronal cell morphology in *Negr1* KO mice. The reductions in total dendritic length, dendritic complexity, and dendritic spine density observed in the NAc neurons of the KO+PBS group were restored in the KO+AKK group following *Akkermansia* administration (Figure 3(d–h), Supplementary Fig. S5A). Additionally, the KO+AKK group showed partial restoration of total dendritic length and complexity, and significant restoration of dendritic spine density in DG neurons (Figure 3(I–m), Supplementary Fig. S5B). In contrast, these neuronal attributes remained unchanged in the WT+AKK group (Figure 3(d–m)). These results suggest that *Akkermansia* administration ameliorates morphological defects in neuronal cells within both the NAc and DG. Taken together, this evidence indicates that frequent exposure to *Akkermansia* spp. alleviates anxiety- and depression-like behaviors in *Negr1* KO mice, potentially by inducing neuronal morphological changes in the NAc and DG.

### Akkermansia administration modulates gene expression in Negr1 knockout mice

**Figure 4.**
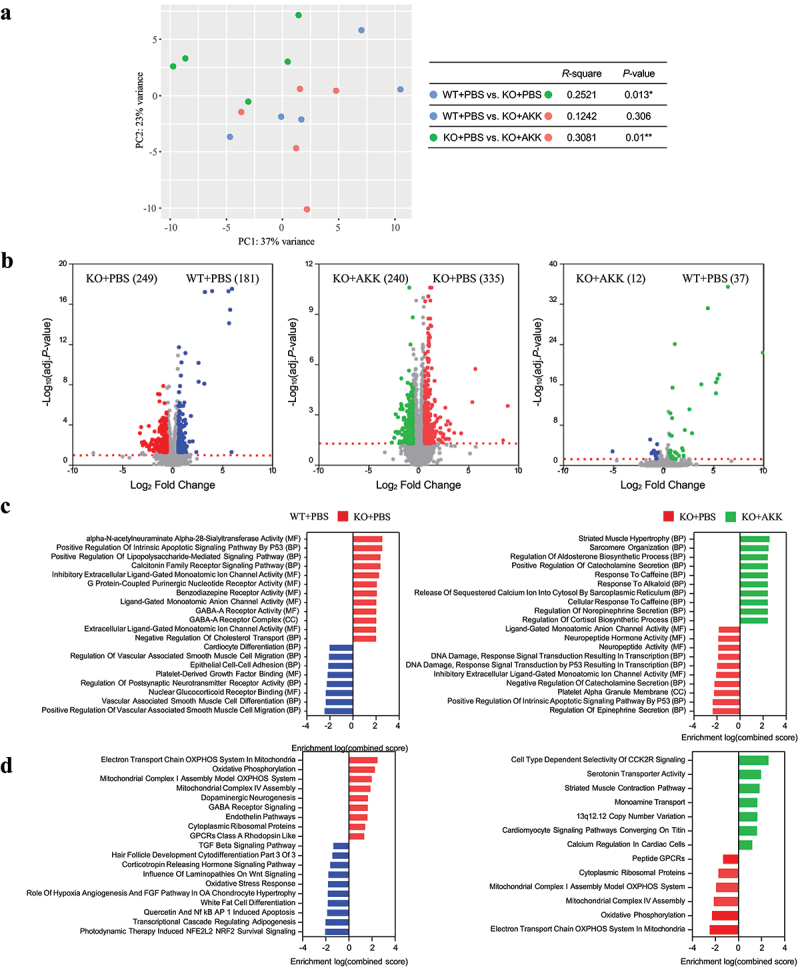
Transcriptome analysis of NAc tissues in WT, *Negr1* KO, and *Negr1* KO mice treated with *Akkermansia* strain.

To understand the mechanisms behind the observed improvements in neuronal morphology and behaviors, we examined the impact of *Akkermansia* administration on gene expression. We conducted RNA-Seq analysis to identify DEGs between the pairs of three groups (WT+PBS, KO+PBS, and KO+AKK) in the brain and colon tissues, aiming to elucidate potential links between genetic alterations and neuronal structures following *Akkermansia* administration. In the NAc of the brain, the gene expression profile of the KO+PBS group significantly differed from those of the WT+PBS (*p* = 0.013) and KO+AKK (*p* = 0.01) groups (Figure 4(a)). Specifically, there was an upregulation of 256 genes and a downregulation of 176 genes in the KO+PBS group compared to the WT+PBS group. Additionally, in the KO+AKK group compared to the KO+PBS group, 239 genes were upregulated and 332 genes downregulated (BH-adjusted *p* < 0.05, fold change > 1.5) (Figure 4(b), Supplementary Fig. S6A). Conversely, the gene expression profiles between the WT+PBS and KO+AKK groups showed no significant differences (*p* = 0.306) (Figure 4(a)), with 37 genes upregulated and 12 genes downregulated (Figure 4(b), Supplementary Fig. S6A). These results suggest that *Akkermansia* treatment reverses the gene expression profile in the NAc of *Negr1* KO mice, making it similar to that of WT mice.

(a) PCA of NAc transcriptomes from WT, *Negr1* KO+PBS, and *Negr1* KO+AKK groups, based on Euclidean distance. *P* value was evaluated using PERMANOVA. (b) Volcano plots displaying differentially expressed genes, showing up-regulated and down-regulated genes in pairwise comparisons among the three groups. (c, d) Functional enrichment analysis with Gene Ontology analysis (c) and WikiPathways analysis (d). They were performed for each pairwise comparison among the three groups. The top 20 features with *p* < 0.01 are displayed.

To gain insights into the biological significance of DEGs, we analyzed functional enrichment in the network using the GO, WikiPathways, and KEGG databases (Figure 4(c,d), Supplementary Fig. S6C). Interestingly, we found that genes involved in the oxidative phosphorylation pathway and aminoacyl tRNA biosynthesis, all of which are mitochondrial genome-encoded, were upregulated in the KO+PBS group compared to the WT+PBS group in the NAc. These same genes were downregulated upon *Akkermansia* treatment (Figure 4(d), Supplementary Fig. S6C). We verified these changes in gene expression by qRT-PCR (Supplementary Fig. S7A). Similar changes were observed in the hippocampus, which includes the dentate gyrus (DG), reinforcing the idea that *Akkermansia*‘s effects on the expression of mitochondrial genome-encoded genes are consistent across different brain regions (Supplementary Fig. S8). In addition, genes related to neuroactive ligand–receptor interaction and response to monoamine neurotransmitters and hormones, such as serotonin and corticosterone, were downregulated in the KO+AKK group compared to KO+PBS (Figure 4(c,d)). We also noted a restoration of the IL-17 signaling pathway, which was depleted in the KO+PBS group (Supplementary Fig. S6C, 7B).

**Figure 5.**
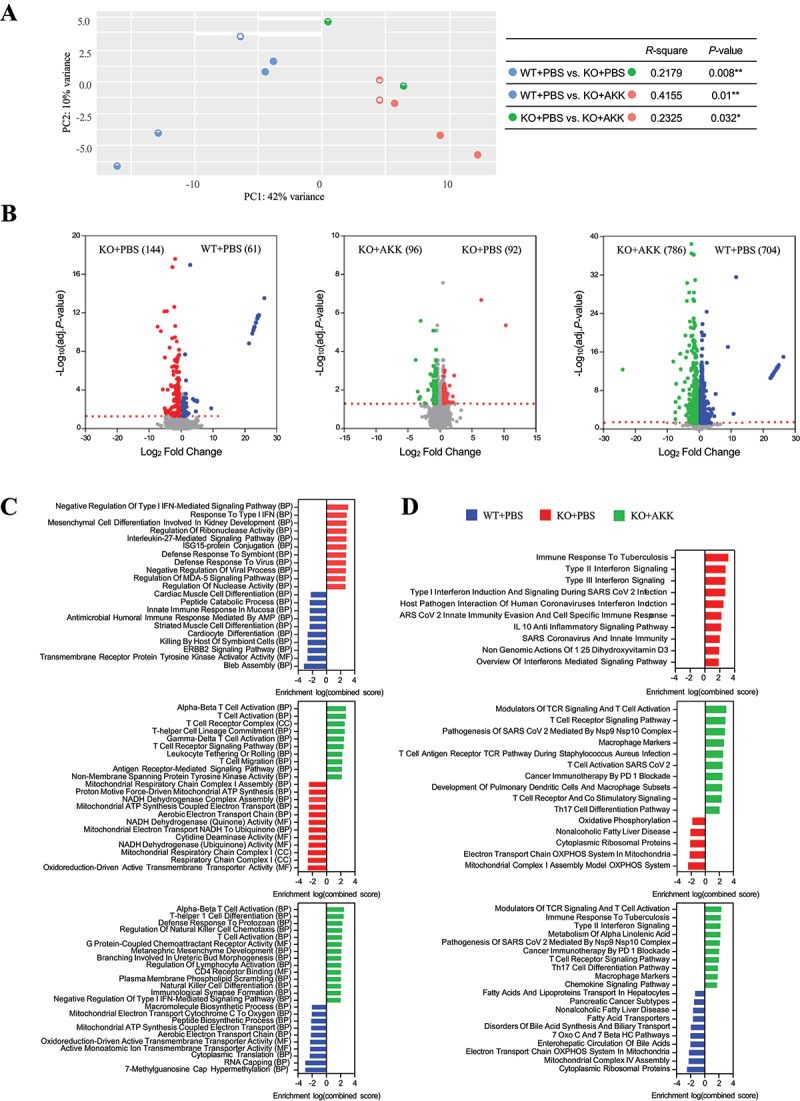
Transcriptome analysis of colon tissues in WT, *Negr1* KO, and *Negr1* KO mice treated with *Akkermansia* strain.

In contrast to the results from the NAc transcriptome, the colonic transcriptome showed significant differences in overall gene expression across all group comparisons (*p* < 0.05), with the KO+AKK group showing the greatest divergence from the WT+PBS group (Figure 5(a)). Specifically, in the KO+PBS group, 144 genes were upregulated and 61 were downregulated compared to the WT+PBS group (Figure 5(b), Supplementary Fig. S6B). Similarly, a comparable number of DEGs were observed between KO+AKK and KO+PBS groups (Figure 5(b)). Notably, the KO+AKK group exhibited a large shift in gene expression, with 786 genes upregulated and 704 downregulated relative to the WT+PBS group (Figure 5(b), Supplementary Fig. S6B). These findings suggest that *Akkermansia* administration induced significant changes in the colonic transcriptome of *Negr1* KO mice, independent of the effects of NEGR1 deficiency.

(a) PCA of colonic transcriptomes from WT, *Negr1* KO+PBS, and *Negr1* KO+AKK groups, based on Euclidean distance. Statistical significance was evaluated using PERMANOVA. (b) Volcano plots displaying differentially expressed genes, showing up-regulated and down-regulated genes in pairwise comparisons among the three groups. (c, d) Gene Ontology (GO) analysis (c) and WikiPathways analysis (d) were performed for each pairwise comparison among the three groups. The top 20 features with *p* < 0.01 are displayed.

We also identified functional enrichment in the colon transcriptome. DEGs upregulated in the KO+PBS group were predominantly associated with interferon-inducible genes, while downregulated genes were enriched for α-defensin, in comparison to the WT+PBS group (Figure 5(c,d), Supplementary Fig. S6D). This suggests that the gut environment of *Negr1* KO mice may be predisposed toward a pro-inflammatory state, supporting the formation of an unstable microbial network (Figure 2(d)). Treatment with *Akkermansia* in *Negr1* KO mice increased the expression of genes associated with T cell signaling, including T cell activation, T cell receptor signaling, and T cell migration.^49^ This upregulation may promote the differentiation of effector T cells, such as Th17 cells, in the KO+AKK group. Additionally, we observed the downregulation of several nuclear-encoded oxidative phosphorylation genes in the KO+AKK group, which contrasts with the mitochondrial genome-encoded genes observed in the NAc transcriptome analysis. These results suggest that *Akkermansia* treatment affects gene expression differentially depending on the tissue.

## Discussion

This study contributes to the understanding of bidirectional interactions between genetic variations and the gut microbiome in the context of depression-like behaviors. The administration of a single strain of *Akkermansia* spp. isolated from WT mice not only alleviated anxiety- and depression-like behaviors in *Negr1* KO mice but also restored impaired dendritic arborization and spine density. The potential mechanisms of *Akkermansia*‘s action are partly hinted by transcriptomic analyses of the NAc, hippocampus, and colonic tissues, raising the possibility that *Akkermansia* may influence mitochondrial function in the brain and immune responses in the colon. These changes could potentially contribute to the observed improvements in both neuronal structure and anxiety- and depression-like behaviors in *Negr1* KO mice.

NEGR1 deficiency in mice induces early-life, community-wide alterations in the gut microbiome, notably characterized by a reduced abundance of *Akkermansia*. In addition to microbiome alterations, *Negr1* KO mice exhibited significant immunological changes in the colon, including the upregulation of IFNγ and IFNγ-inducible genes, such as *Irgm1*, alongside a downregulation of antimicrobial peptides like α-defensin. These findings align with a previous report that IFNγ induction impairs the secretion of antimicrobial peptides from Paneth cells by depleting antimicrobial peptide-containing granules.^50^ This mechanistic link is further substantiated by finding that *Akkermansia* abundance increases significantly in IFNγ-deficient mice but decreases upon IFNγ restoration.^42^ Therefore, the tripartite relationship between IFNγ, antimicrobial peptides, and *Akkermansia* likely contributes to the observed microbiota alterations in *Negr1* KO mice.

The role of *A. muciniphila* in maintaining gut barrier integrity and its association with various health issues, such as intestinal inflammation, obesity, type 2 diabetes, and liver steatosis, have been established.^41,51–53^ Recently, its potential involvement in neurological disorders is gaining attention, with studies noting a decrease in the abundance of *A. muciniphila* in conditions like amyotrophic lateral sclerosis, Alzheimer’s disease, autism spectrum disorder, and depression models induced by chronic stress or antibiotics.^54–58^ However, the causative role of *A. muciniphila* has not been well established in most studies with neurological disorders, with only a few studies addressing this issue. In depression, oral treatment of *A. muciniphila* elicits anti-depressive effects from a mouse model subjected to either chronic restraint stress or antibiotic treatment.^59–61^ Postnatal supplementation of *A. muciniphila* ameliorates valproic acid-induced social deficits in mice.^62^ In contrast to these models, our study demonstrates that *Akkermansia* administration ameliorated depression-like behaviors in a genetically predisposed mouse model with NEGR1 deficiency. Furthermore, our study importantly uncovered the new trait of *A. muciniphila* influencing neuronal cell structures, specifically its ability to restore impaired dendritic arborization caused by NEGR1 deficiency. This finding partly aligns with the previous study reporting the restorative effect of *A. muciniphila* in the degeneration of motor neurons in the *Sod1*-Tg mouse model of ALS.^54^ Overall, it seems likely that gut-resident *A. muciniphila* can manipulate neuronal functions in the brain and peripheral organs. Further investigations are needed to clarify the neuro-modulatory effects of gut-associated *A. muci niphila* in other neuronal disease models.

Several studies have proposed mechanisms by which *A. muciniphila* may influence stress-induced depression.^57,59^ One proposed mechanism is the suppression of neuroinflammation, which contributes to neurodegeneration.^63^ *A. muciniphila* activates TLR2 signaling and reduces pro-inflammatory factors such as TNFα, IL-1, and IL-6.^64,65^ Notably, in a chronic stress-induced depression mouse model, the outer membrane protein of *A. muciniphila* Amuc_1100 was shown to suppress neuroinflammation by activating TLR2 signaling and decreasing the levels of these pro-inflammatory cytokines.^66,67^ However, our transcriptomic analysis revealed modulation of IL-17-related pathways, suggesting that *Akkermansia* may influence neuronal function and behavior in *Negr1* KO mice through an alternative neuroimmune mechanism. IL-17 signaling has been implicated in neuroimmune interactions, synaptic plasticity, and neuroinflammation,^68,69^ but its specific role in *Akkermansia*-mediated effects remains to be elucidated. Further studies are required to investigate whether IL-17 modulation contributes to the observed behavioral and neuronal improvements.

Another potential mechanism involves the regulation of brain-derived neurotrophic factor (BDNF), which promotes neurogenesis, dendritogenesis, synaptogenesis, and neuronal survival by activating the TrkB receptor.^58,66,70,71^ Previous studies have suggested that *Akkermansia* spp. may modulate BDNF expression^70,72^; however, *Negr1* KO mice did not exhibit significant changes in BDNF mRNA expression in the hippocampus or whole brain,^24^ and our RNA-seq analysis confirmed no significant differences in hippocampal BDNF expression among the WT+PBS, KO+PBS, and KO+AKK mice. These results suggest that the improvement in anxiety- and depression-like behaviors of *Negr1* KO mice following *Akkermansia* administration may not be directly related to BDNF regulation.

Beyond their traditional role as cellular powerhouses, mitochondria are emerging as key regulators of neuronal structure, function, and behavior. Mitochondrial dysfunction has increasingly been linked to neuropsychiatric disorders,^73,74^ which often exhibit structural changes in neurons, such as dendritic atrophy and spine loss.^75,76^ Recent evidence further supports a strong link between mitochondrial dynamics and psychiatric conditions, including anxiety and depression.^14,77^ For instance, high-anxiety animal models show reduced mitofusin 2 levels specifically in medium spiny neurons of NAc, leading unexpectedly to larger, rounded mitochondria rather than the smaller, fragmented structures typically associated with impaired fusion. Moreover, these neurons exhibit fewer mitochondria-endoplasmic reticulum contacts, challenging conventional assumptions about mitochondrial division,^14^ suggesting that mitochondrial regulation is highly cell-type-specific. Given the critical role of mitochondria in neuronal health, our findings raise the possibility that mitochondria-related processes might be involved in the behavioral and structural abnormalities observed in *Negr1* KO mice. Our transcriptomic analyses revealed significant alterations, particularly an upregulation of mitochondrial genome-encoded genes in the examined brain regions in *Negr1* KO mice (Figure 4d, Supplementary Figs. S6C and S7A). Previous studies have shown that metabolic stress, such as high glucose-induced reactive oxygen species (ROS), can trigger a compensatory increase in mtDNA-encoded electron transport subunit expression.^78^ Since NEGR1 deficiency is associated with obesity and Type II diabetes,^79^ metabolic stress may similarly influence mitochondrial genome-encoded gene expression in *Negr1* KO mice. Interestingly, Wang *et al*. recently reported that *Akkermansia muciniphila* may influence mitochondrial homeostasis, potentially through the production of short-chain fatty acids (SCFAs), such as propionic acid.^80^ However, in our study, no significant differences in cecal concentrations of SCFAs, including acetate, propionate, and butyrate, were observed among the WT+PBS, KO+PBS, and KO+AKK mice (Supplementary Fig. S9), suggesting that the observed mitochondrial gene expression changes and beneficial effects of *Akkermansia* are likely independent of SCFAs modulation.

*A. muciniphila* has been shown to exhibit diverse immunomodulatory effects depending on the disease model. It can promote regulatory T cells in the adipose tissue and the colon in models of diet-induced obesity and colitis^38,81,82^ while modulating CD8^+^ T lymphocyte activation in cancer models,^83^ suggesting that *Akkermansia* has a flexible role in immune regulation, adapting to different physiological contexts. In *Negr1* KO mice, the gut appeared predisposed toward a pro-inflammatory state (Figure 5c,d), resulting in an unstable microbial network (Figure 2d). Treatment with the *Akkermansia* strain modulated this gut environment, increasing the expression of genes involved in the T cell receptor signaling pathway (*Cd247, Cd3e, Lck, Zap70, Lat, Itk*, and *Pik3cd*) in the colon (Figure 5c,d). These findings suggest that *Akkermansia*’s immunomodulatory properties restored the skewed immune homeostasis caused by NEGR1 deficiency.

One such pathway involves IL-17, a pro-inflammatory cytokine produced mainly by Th17 cells, which plays a complex role in both health and disease states.^84^ Chronic IL-17 activity is involved in the pathogenesis of inflammatory diseases, such as psoriasis, rheumatoid arthritis, and inflammatory bowel disease, while it also contributes to mucosal immunity by promoting antimicrobial peptide production and maintaining epithelial barrier integrity in the intestine.^84,85^ Similarly, the role of IL-17 in mood and behavior is paradoxical. Elevated blood IL-17 levels are observed in patients with depression,^86–88^ and chronic stress-induced IL-17 contributes to depression-like behaviors through neuroinflammation in mice.^89^ Conversely, IL-17 deficiency has been associated with social avoidance behaviors,^90,91^ suggesting that an appropriately balanced IL-17 response is crucial for normal neuroimmune function. Notably, the NAc of *Negr1* KO mice showed low expression of IL-17 signaling genes (*Il17ra, Traf5*) compared to WT, but *Akkermansia* treatment reversed this trend by upregulating these same genes (Supplementary Fig. S7B). This points to a broader immunoregulatory effect of *Akkermansia* that extends beyond the peripheral immunity into central neuroimmune circuits, potentially affecting both behavior and overall inflammatory status.

*Akkermansia* has also been shown to improve metabolic dysfunctions by enhancing intestinal barrier integrity.^92^ To investigate whether NEGR1 deficiency impairs gut barrier function or whether *Akkermansia* spp. could improve it in *Negr1* KO mice, we assessed mucus thickness and related markers. However, no significant differences in mucus thickness were observed (Supplementary Fig. S10A, 10B). Furthermore, *Akkermansia* treatment did not significantly affect the expression of genes encoding mucin (MUC2) or tight junction proteins (ZO-1 and occludin) (Supplementary Fig. S10C).

In conclusion, our study uncovers a critical interaction between host genetics and the gut microbiome in modulating neuropsychiatric phenotypes associated with NEGR1 deficiency (Supplementary Fig. S11). We found that NEGR1 deficiency leads to alterations in gut microbiome composition, specifically reducing early-life abundance of *Akkermansia* spp., which is causally linked to neuronal structural abnormalities and depression-like behaviors in *Negr1* KO mice. Remarkably, *Akkermansia* spp. administration induced substantial changes in gene expression in both the brain and gut, including the expression of genes associated with mitochondrial function and immune homeostasis. These findings support a potential bidirectional relationship in which host genetic predispositions shape gut microbiome composition, which in turn modulates host gene expression and neuronal integrity. While our findings strongly suggest that *Akkermansia* plays a key role, we acknowledge that other microbial components may also contribute to these phenotypes. Understanding how specific microbial taxa and their interactions shape neuronal function may provide new insights into microbiome-based interventions for neuropsychiatric disorders, particularly in individuals with genetic predispositions to altered gut-brain axis regulation.

## Materials and methods

### Animals

All animal studies were approved by the Institutional Animal Care and Use Committee of Chungnam National University (202012A-CNU-177). Male C57BL/6J wild-type (WT) and *Negr1* KO (*Negr1*^−/−^) mice^24^ were used in this study. The mice were housed in groups of 3–5 per cage under a 12-h light–dark cycle with *ad libitum* access to food and water.

### Fecal 16S rRNA gene amplicon analysis

Metagenomic DNA was extracted from fecal samples (0.05–0.1 g) using the QIAamp Fast DNA Stool Mini Kit (Qiagen, Germany), as described in the protocol Q of Costea et al.^93^ Repeated bead beating was performed at 5.5 m/s for 30 s using the FastPrep®-24 instrument (MP Biomedicals, USA), as described by Zoetendal *et al*.^94^ The V3-V4 region of the 16S rRNA gene was amplified using primers 341F and 805R combined with Illumina adapters. Amplicons (3–5 replicates per sample) were pooled and purified with the QIAquick PCR Purification Kit (Qiagen, Germany). Sequencing was performed using the Illumina MiSeq platform (2 × 300 bp), according to the manufacturer’s instructions (Illumina, USA). The 16S rRNA gene sequences were processed using QIIME2 (version 2018.11), as described previously.^95^ Briefly, primer sequences were trimmed from paired-end raw reads, and Divisive Amplicon Denoising Algorithm 2^36^ was used to generate amplicon sequence variants (ASVs) after filtering out low-quality reads, correcting errors, removing chimeric sequences, and merging into a single read. Samples were rarefied evenly to 40,000 sequences, and ASVs were removed when the following conditions were met: 1) fewer than 40 sequences, and 2) observed in less than six samples. Taxonomic classification was performed using a Naive Bayesian classifier trained on the de-replicated sequences (99% similarity) of Greengenes database (2013–08 release). Diversity matrices (Observed_OTUs, Shannon index, and Pielou’s evenness) were estimated. Beta-diversity was determined based on Bray-Curtis dissimilarity and Jaccard distance. Spearman correlations among ASVs (>0.1% of average abundance) were calculated within groups using CCREPE.^96^ Microbial association networks were constructed from significant positive and negative correlations (*r* > 0.4, *p* < 0.01) and visualized with Cytoscape 3.8.2 using the Compound Spring Embedder Layout. The modules of co-occurrence network were identified using the Clauset – Newman – Moore algorithm (*greedy_modularity_communities* from the *networkx* Python package),^97^ with modularity calculated via *nx_comm.modularity*. The ratio of negative-to-positive association was calculated manually.

### Cultural isolation of Akkermansia strains

Cultural strains of *Akkermansia* spp. were isolated from fecal samples of WT mice. Fecal samples were suspended in 1 ml of anaerobic phosphate-buffered saline (PBS, Bioneer, South Korea) containing 0.05% L-cysteine hydrochloride (Sigma, USA). Suspensions were cultured on brain heart infusion medium without dextrose (Kisan-Bio, South Korea), supplemented with 0.4% type III porcine stomach mucin (BHI-M, Sigma, USA), and on BHI-M added with 5 μg/ml vancomycin (Sigma, USA). Cultures were incubated for 72 h at 37°C in an anaerobic chamber (COY Laboratory Products, USA), following a previously established protocol.^40^ The isolates were sub-cultured in the same medium, and 16S rRNA gene sequences were amplified by colony PCR using universal primers 27F and 1492R. The PCR products were purified with a QIAquick PCR Purification kit (Qiagen, Germany) and sequenced using a BigDye Terminator Cycle Sequencing Ready Reaction kit (Applied Biosystems, USA), according to the manufacturers’ instructions. The sequencing reaction products were analyzed using the PRISM 3730XL DNA Analyzer (Applied Biosystems, USA). Partial 16S rRNA gene sequences were assembled using SeqMan (DNASTAR, USA) and compared to sequences in the NCBI nr database via BLASTn. The V3-V4 region sequences of the isolates were aligned with 16S rRNA gene sequences of *Akkermansia* ASVs, *A*. *muciniphila* Muc^T^, *A*. *muciniphila* GP strains, *A*. *glycaniphila* Pyt^T^, *A*. *muciniphila* EB-AMDK, *A*. *muciniphila* JCM 30,893, and *A*. *muciniphila* Akk, using Clustal Omega from the EMBL-EBI. The trimmed alignment was converted to MEGA format for phylogenetic analyses. Phylogenetic consensus trees were constructed using the maximum-likelihood method with MEGA X and evaluated using 1,000 bootstrap replicates.

### Genotyping of Akkermansia strains isolates

Genomic DNA was extracted from ten isolates and the type strain of *A. muciniphila* Muc^T^ (DSM 22,959) using the QIAamp Fast DNA Stool Mini Kit (Qiagen, Germany) following the manufacturer’s instructions. ERIC-PCR was performed with ERIC primers (ERIC-1 5’-ATG TAA GCT CCT GGG GAT TCA C-3’; ERIC-2 5’-AAG TAA GTG ACT GGG GTG AGC G-3’), as previously described.^98^ A 10 μl aliquot of PCR product was stained with an equal volume of 1× loading star dye (Dyne Bio, South Korea) and loaded onto 1.5% agarose gel. Electrophoresis was performed at 50 V for 1 h and photographed under UV light.

### Oral administration of an Akkermansia isolate

*Akkermansia* spp. strain m3-2 was cultured on and harvested from BHI-M medium and suspended in PBS containing 0.05% L-cysteine hydrochloride. Fresh cell suspensions of strain m3-2 were prepared weekly, stored at 4°C, and pre-warmed to room temperature before administration. Four groups of 6–8-wk-old mice received oral administration of 200 μl of pre-reduced PBS or the m3-2 cell suspension every weekday for 4 wk. The cell suspension contained an average of 9.3 ± 0.3 log colony-forming units (CFU) per 200 μl, based on CFU counts on BHI-M medium (Supplementary Fig. S3B). To ensure stability in cell count and viability during 4°C storage, we compared the viable cell counts of strain m3-2 before and after storage, confirming no significant changes (Supplementary Fig. S3C).

### Golgi-Cox staining

Golgi-Cox staining was performed following a previously described protocol.^99^ After behavioral tests, mice were decapitated, and brains were rapidly rinsed in double-distilled water (dd-H_2_O). Brains were then immersed in Golgi-Cox solution (1.04% potassium dichromate, 1.04% mercuric chloride, and 0.83% potassium chromate) for 7–10 d before being transferred to a tissue protectant solution (0.05 M phosphate buffer with 30% sucrose and 30% ethylene glycol). Coronal brain sections, 150 μm thick, were cut using a vibratome (VT1000S, Leica, Germany) and mounted on microscope slides with the tissue protectant solution. After air drying for 3 d, sections were developed. Sections were first incubated in 50% ethanol for 5 min, then in 66.6% ammonia solution for 8 min, followed by rinsing with dd-H_2_O. Sections were then treated with 5% sodium thiosulfate for 10 min in the dark, rinsed with dd-H_2_O, and dehydrated in graded ethanol solutions (70%, 95%, and 100%) for 5 min each. Finally, sections were cleared in xylene and mounted with Permount (SP15–100, Fisher Chemical, UK).

### Neurostructural analysis

Neurostructural images were acquired using a confocal microscope (LSM 880, Carl Zeiss, Germany) with a 20× objective (2048 × 2048 pixels; 1 μm z-stack intervals). Golgi-stained neurons were traced in 3D using Neurolucida software (MBF Bioscience), allowing for detailed reconstructions of dendritic structures. Total dendritic length and complexity were analyzed with NeuroExplorer software (MBF Bioscience, USA). To quantify spine density, three dendritic segments (secondary or tertiary dendrites >30 μm in length) per neuron were selected, and all dendritic protrusions were counted using ImageJ software (NIH, USA). Counts were averaged for each neuron.

### Three-chamber sociability test

The 3-chamber test was performed according to a previously described procedure.^100^ The apparatus consisted of a rectangular box divided into three connected compartments, each separated by transparent walls. Wire cages were placed in the compartments flanking the central arena. Before the test, the subject mouse was habituated to the apparatus for 10 min. In the test, the mouse was placed in the center compartment with access to the connecting chambers blocked. A stimulus mouse, designated as “Stranger 1”, was randomly placed in a wire cage. When the chambers were unblocked, the subject mouse could explore the entire apparatus freely. The test lasted 10 min, during which the mouse’s movements were recorded for sociability assessment. Time spent in each zone was analyzed using ANY-maze software (Stoelting Co., USA).

### Elevated plus maze test

The elevated plus maze test was conducted according to a previously described procedure.^101^ The apparatus consisted of two opposite rectangular arms without walls, designated as “open arms” (25 cm × 5 cm), two opposite rectangular arms with opaque walls, designated as “closed arms” (25 cm × 5 cm × 16 cm), connected by a central square platform (5 cm × 5 cm). The plus maze was elevated 50 cm above the floor. Each mouse was placed in the center of the maze and recorded for 5 min, during which time spent in the open and closed arms was analyzed using ANY-maze software (Stoelting Co., USA).

### Forced swimming test

The forced swimming test was conducted following an established procedure.^102^ Mice were placed individually in a clear glass cylinder (20 cm diameter, 20 cm height) filled with water (25 ± 1°C) to a depth of 12 cm. Water was replaced after each trial. Each mouse was gently placed in the cylinder and recorded for a total of 6 min. Immobility was defined as the time during which the mouse ceased swimming and floated motionless at the water surface. Immobility time was analyzed during the final 4 min of the test.

### RNA extraction and quantitative real-time PCR

Total RNA was extracted from colonic tissue using TRIzol® reagent (Invitrogen, USA), and complementary DNA (cDNA) was synthesized using a cDNA Synthesis Kit (TOYOBO, Japan). The cDNA was then used as a template for quantitative real-time PCR, conducted with the SYBR Green Real-Time PCR Master Mix Kit (TOYOBO, Japan). Gene expression levels were normalized to β-actin mRNA levels and expressed as fold induction, calculated using the 2^−ΔΔCT^ method.

### Transcriptome analysis

WT (WT+PBS), *Negr1* KO (KO+PBS), and *Negr1* KO mice treated with *Akkermansia* (KO+AKK) (n = 5 per group) were sacrificed by decapitation, and brains and entire colons were quickly dissected. The brains were rinsed briefly with ice-cold PBS and immediately frozen in liquid nitrogen. Each brain was sectioned into 1 mm slices using a coronal brain matrix (L.*R*-68707, L.M.S., South Korea), and the NAc and DG regions were isolated with a 1 mm biopsy punch (BPP-10F, Kai Medical, Japan) on dry ice. Colons were stored in cold PBS, and the proximal segments (~1 cm) were preserved in RNAlater solution (Invitrogen, USA). Total RNA was extracted from brain and colonic tissues using the RNA purification kit (Macherey-Nagel, Germany). RNA-sequencing libraries were prepared using the TruSeq Stranded RNA Sample Preparation Kit (Illumina, USA). Sequencing was performed on the Illumina NovaSeq X platform with paired-end reads. Raw reads were processed to remove low-quality reads, adapter sequences, and PCR duplicates. Trimmed reads were mapped to the mouse reference genome (mm10) using HISAT2, and aligned reads were assembled and quantified using StringTie. Differential expression analysis was conducted with DESeq2^103^ or PyDESeq2.^104^ Genes with a Benjamini-Hochberg adjusted *p*-value <0.1 and a fold-change > ±1.5 were considered differentially expressed (DEGs) between groups. Regularized log-transformed expression values were compared based on Euclidean distance and visualized with principal component analysis (PCA). Gene set enrichment analysis was performed on DEGs against Gene Ontology, WikiPathways, and KEGG databases using Enrichr.^105^

### Measurement of mucus thickness

Mesenteric fat tissue was removed from colons and flushed with ice-cold 1× PBS to clear luminal contents. For Periodic acid-Schiff (PAS) staining, a 1 cm section of the colon was fixed in Carnoy’s solution (60% methanol, 30% chloroform, 10% acetic acid). Fixed colon segments were embedded in Tissue-Tek O.C.T. compound (SAKURA, Japan), sectioned at 10 μm thickness, and stained with PAS for mucus layer measurement. Mucus thickness was measured at 50 points within the inner mucus layer using ImageJ software (NIH, USA).

### Measurement of cecal SCFAs

Approximately 200 mg of cecal contents were used for acetate, propionate, butyrate, valerate, isobutyrate, and isovalerate concentrations. Frozen cecal samples were homogenized in five volumes of dH_2_O and centrifuged at 13,000 rpm for 10 min at 4°C. The 150 µL of supernatants were transferred to 10 mL of screw-cap vials containing 150 µL of buffer solution with (NH_4_)_2_SO_4_, NaH_2_PO_4_, and 2-ethylbutyric acid as an internal standard. Headspace sampler-gas chromatography-Flame ionization detector (HS-GS-FID) analysis was performed by Agilent 7890B GC system equipped with a 7697A headspace sampler and FID (Agilent Technologies, USA). An HP-innowax capillary column (30 m × 250 µm × 0.25 µm film thickness; Agilent) was used with nitrogen as the carrier gas at a constant flow rate. The operating conditions were as follows: oven temperature, 85°C; loop temperature 90°C; transfer lines, 100°C; FID temperature 250°C; column temperature was initially at 60°C, raised to 140°C at 30°C/min, then raised to 170°C at 30°C, and finally to 180°C at 40°C and held for 0.75 min. Data acquisition and operation processing were conducted using ChemStation software (Agilent Technologies). SCFA levels were identified and quantified using standard compounds.

### Statistical analysis

All graphing and statistical analyses were performed using GraphPad Prism version 5.01 and 10.3.0 for Windows (GraphPad Software, USA). The Shapiro–Wilk normality test was performed to assess whether the data sets were normally distributed. Depending on the data characteristics, paired or unpaired two-tailed *t*-tests, one-way ANOVA, and two-way ANOVA were used with a significance set at *p* < 0.05. Two-tailed Mann–Whitney U tests were also performed. Principal Coordinates Analysis (PCoA) was performed based on Bray-Curtis dissimilarity and Jaccard distance using R package ‘vegan’.^106^ Statistical significances for PCoA analysis were assessed using the ‘adonis’ function with 999 permutations. The percentages of explained variances for environmental variables were calculated based on Bray-Curtis dissimilarity using distance-based redundancy analysis (db-RDA) of R package ‘vegan’.^106^ The relative abundance of ASVs was correlated with the first axis of the db-RDA constrained ordination using the ‘add.spec.scores’ function from the ‘BiodiversityR’ package. Permutation tests were performed using the ‘envfit’ function in ‘vegan’ with 999 permutations, and ASVs discriminating between groups were identified using LEfSe analysis.^39^

## Supplementary Material

Suppl_Figures_Revision_JYKIM.docx

## Data Availability

The 16S rRNA amplicon and transcriptome data were deposited in the European Nucleotide Archive under the accession number PRJEB79790.

## References

[1] Hasler G. Pathophysiology of depression: do we have any solid evidence of interest to clinicians? World Psychiatry. 2010;9(3):155–24. doi: 10.1002/j.2051-5545.2010.tb00298.x. 20975857 PMC2950973

[2] Winter G, Hart RA, Charlesworth RPG, Sharpley CF. Gut microbiome and depression: what we know and what we need to know. Rev Neurosciences. 2018;29(6):629–643. doi: 10.1515/revneuro-2017-0072. 29397391

[3] Li Y, Li J, Cheng R, Liu H, Zhao Y, Liu Y, Chen Y, Sun Z, Zhai Z, Wu M, et al. Alteration of the gut microbiome and correlated metabolism in a rat model of long-term depression. Front Cell Infect Microbiol. 2023;13:1116277. doi: 10.3389/fcimb.2023.1116277. 37051300 PMC10084793

[4] Zhou M, Fan Y, Xu L, Yu Z, Wang S, Xu H, Zhang J, Zhang L, Liu W, Wu L, et al. Microbiome and tryptophan metabolomics analysis in adolescent depression: roles of the gut microbiota in the regulation of tryptophan-derived neurotransmitters and behaviors in human and mice. Microbiome. 2023;11(1):145. doi: 10.1186/s40168-023-01589-9. 37386523 PMC10311725

[5] Jiang H, Ling Z, Zhang Y, Mao H, Ma Z, Yin Y, Wang W, Tang W, Tan Z, Shi J, et al. Altered fecal microbiota composition in patients with major depressive disorder. Brain Behav Immun. 2015;48:186–194. doi: 10.1016/j.bbi.2015.03.016. 25882912

[6] Naseribafrouei A, Hestad K, Avershina E, Sekelja M, Linlokken A, Wilson R, Rudi K. Correlation between the human fecal microbiota and depression. Neurogastroenterol Motil: Off J Eur Gastrointestinal Motil Soc. 2014;26(8):1155–1162. doi: 10.1111/nmo.12378. 24888394

[7] Chen MM, Wang P, Xie XH, Nie Z, Xu SX, Zhang N, Wang W, Yao L, Liu Z. Young adults with Major depression show altered microbiome. Neuroscience. 2023;522:23–32. doi: 10.1016/j.neuroscience.2023.05.002. 37169166

[8] Bharwani A, Mian MF, Foster JA, Surette MG, Bienenstock J, Forsythe P. Structural & functional consequences of chronic psychosocial stress on the microbiome & host. Psychoneuroendocrinology. 2016;63:217–227. doi: 10.1016/j.psyneuen.2015.10.001. 26479188

[9] Kelly JR, Borre Y, Ob C, Patterson E, El Aidy S, Deane J, Kennedy PJ, Beers S, Scott K, Moloney G, et al. Transferring the blues: depression-associated gut microbiota induces neurobehavioural changes in the rat. J Psychiatric Res. 2016;82:109–118. doi: 10.1016/j.jpsychires.2016.07.019. 27491067

[10] Zheng P, Zeng B, Zhou C, Liu M, Fang Z, Xu X, Zeng L, Chen J, Fan S, Du X, et al. Gut microbiome remodeling induces depressive-like behaviors through a pathway mediated by the host’s metabolism. Mol Psychiatry. 2016;21(6):786–796. doi: 10.1038/mp.2016.44. 27067014

[11] Wohleb ES, Franklin T, Iwata M, Duman RS. Integrating neuroimmune systems in the neurobiology of depression. Nat Rev Neurosci. 2016;17(8):497–511. doi: 10.1038/nrn.2016.69. 27277867

[12] Klawonn AM, Malenka RC. Nucleus accumbens modulation in reward and aversion. Cold Spring Harbor Symposia On Quant Biol. 2018;83:119–129. doi: 10.1101/sqb.2018.83.037457. PMC6650377 30674650

[13] Fox ME, Chandra R, Menken MS, Larkin EJ, Nam H, Engeln M, Francis TC, Lobo MK. Dendritic remodeling of D1 neurons by RhoA/Rho-kinase mediates depression-like behavior. Mol Psychiatry. 2020;25(5):1022–34. doi: 10.1038/s41380-018-0211-5. 30120419 PMC6378138

[14] Gebara E, Zanoletti O, Ghosal S, Grosse J, Schneider BL, Knott G, Astori S, Sandi C. Mitofusin-2 in the nucleus Accumbens regulates anxiety and depression-like behaviors through mitochondrial and neuronal actions. Biol Psychiatry. 2021;89(11):1033–1044. doi: 10.1016/j.biopsych.2020.12.003. 33583561

[15] Guzman SJ, Schlögl A, Espinoza C, Zhang X, Suter BA, Jonas P. How connectivity rules and synaptic properties shape the efficacy of pattern separation in the entorhinal cortex–dentate gyrus–CA3 network. Nat Comput Sci. 2021;1(12):830–842. doi: 10.1038/s43588-021-00157-1. 38217181

[16] Umschweif G, Greengard P, Sagi Y. The dentate gyrus in depression. Eur J Neurosci. 2021;53(1):39–64. doi: 10.1111/ejn.14640. 31811730

[17] Sahay A, Drew MR, Hen R. Dentate gyrus neurogenesis and depression. Prog Brain Res. 2007;163:697–722. 10.1016/S0079-6123(07)63038-6. 17765746

[18] Agrimi J, Bernardele L, Sbaiti N, Brondi M, D’Angelo D, Canato M, Marchionni I, Oeing CU, Barbara G, Vignoli B, et al. Reiterated male-to-female violence disrupts hippocampal estrogen receptor β expression, prompting anxiety-like behavior. iScience. 2024;27(9):110585. doi: 10.1016/j.isci.2024.110585. 39228787 PMC11369378

[19] Luczynski P, Whelan SO, O’Sullivan C, Clarke G, Shanahan F, Dinan TG, Cryan JF. Adult microbiota-deficient mice have distinct dendritic morphological changes: differential effects in the amygdala and hippocampus. Eur J Neurosci. 2016;44(9):2654–2666. doi: 10.1111/ejn.13291. 27256072 PMC5113767

[20] Singh K, Jayaram M, Kaare M, Leidmaa E, Jagomae T, Heinla I, Hickey MA, Kaasik A, Schäfer MK, Innos J, et al. Neural cell adhesion molecule Negr1 deficiency in mouse results in structural brain endophenotypes and behavioral deviations related to psychiatric disorders. Sci Rep. 2019;9(1):5457. doi: 10.1038/s41598-019-41991-8. 30932003 PMC6443666

[21] Pischedda F, Piccoli G. The IgLON family member Negr1 promotes neuronal arborization acting as soluble factor via FGFR2. Front Mol Neurosci. 2015;8:89. doi: 10.3389/fnmol.2015.00089. 26793057 PMC4710852

[22] Sanz R, Ferraro GB, Fournier AE. IgLON cell adhesion molecules are shed from the cell surface of cortical neurons to promote neuronal growth. J Biol Chem. 2015;290(7):4330–4342. doi: 10.1074/jbc.M114.628438. 25538237 PMC4326840

[23] Szczurkowska J, Pischedda F, Pinto B, Manago F, Haas CA, Summa M, Bertorelli R, Papaleo F, Schäfer MK, Piccoli G, et al. NEGR1 and FGFR2 cooperatively regulate cortical development and core behaviours related to autism disorders in mice. Brain. 2018;141(9):2772–2794. doi: 10.1093/brain/awy190. 30059965 PMC6113639

[24] Noh K, Lee H, Choi TY, Joo Y, Kim SJ, Kim H, Kim JY, Jahng JW, Lee S, Choi S-Y, et al. Negr1 controls adult hippocampal neurogenesis and affective behaviors. Mol Psychiatry. 2019;24(8):1189–1205. doi: 10.1038/s41380-018-0347-3. 30651602

[25] Hyde CL, Nagle MW, Tian C, Chen X, Paciga SA, Wendland JR, Tung JY, Hinds DA, Perlis RH, Winslow AR. Identification of 15 genetic loci associated with risk of major depression in individuals of European descent. Nat Genet. 2016;48(9):1031–1036. doi: 10.1038/ng.3623. 27479909 PMC5706769

[26] Wray NR, Ripke S, Mattheisen M, Trzaskowski M, Byrne EM, Abdellaoui A, Adams MJ, Agerbo E, Air TM, Andlauer TMF, et al. Genome-wide association analyses identify 44 risk variants and refine the genetic architecture of major depression. Nat Genet. 2018;50(5):668–681. doi: 10.1038/s41588-018-0090-3. 29700475 PMC5934326

[27] Howard DM, Adams MJ, Clarke TK, Hafferty JD, Gibson J, Shirali M, Coleman JRI, Hagenaars SP, Ward J, Wigmore EM, et al. Genome-wide meta-analysis of depression identifies 102 independent variants and highlights the importance of the prefrontal brain regions. Nat Neurosci. 2019;22(3):343–352. doi: 10.1038/s41593-018-0326-7. 30718901 PMC6522363

[28] Dall’aglio L, Lewis CM, Pain O. Delineating the genetic component of gene expression in Major depression. Biol Psychiatry. 2021;89(6):627–636. doi: 10.1016/j.biopsych.2020.09.010. 33279206 PMC7886308

[29] Duric V, Banasr M, Licznerski P, Schmidt HD, Stockmeier CA, Simen AA, Newton SS, Duman RS. A negative regulator of MAP kinase causes depressive behavior. Nat Med. 2010;16(11):1328–1332. doi: 10.1038/nm.2219. 20953200 PMC3066515

[30] Barreto RA, Walker FR, Dunkley PR, Day TA, Smith DW. Fluoxetine prevents development of an early stress-related molecular signature in the rat infralimbic medial prefrontal cortex. Implic For Depression? BMC Neurosci. 2012;13:125. 10.1186/1471-2202-13-125. PMC3528467 23075086

[31] Singh K, Loreth D, Pottker B, Hefti K, Innos J, Schwald K, Hengstler H, Menzel L, Sommer CJ, Radyushkin K, et al. Neuronal growth and behavioral alterations in mice deficient for the psychiatric disease-associated Negr1 gene. Front Mol Neurosci. 2018;11:30. doi: 10.3389/fnmol.2018.00030. 29479305 PMC5811522

[32] LeGates TA, Kvarta MD, Tooley JR, Francis TC, Lobo MK, Creed MC, Thompson SM. Reward behaviour is regulated by the strength of hippocampus–nucleus accumbens synapses. Nature. 2018;564(7735):258–262. doi: 10.1038/s41586-018-0740-8. 30478293 PMC6292781

[33] Morales-Medina JC, Sanchez F, Flores G, Dumont Y, Quirion R. Morphological reorganization after repeated corticosterone administration in the hippocampus, nucleus accumbens and amygdala in the rat. J Chem Neuroanat. 2009;38(4):266–272. doi: 10.1016/j.jchemneu.2009.05.009. 19505571

[34] Bosch JA, Nieuwdorp M, Zwinderman AH, Deschasaux M, Radjabzadeh D, Kraaij R, Davids M, de Rooij SR, Lok A. The gut microbiota and depressive symptoms across ethnic groups. Nat Commun. 2022;13(1):7129. doi: 10.1038/s41467-022-34504-1. 36473853 PMC9726934

[35] Radjabzadeh D, Bosch JA, Uitterlinden AG, Zwinderman AH, Ikram MA, van Meurs JBJ, Luik AI, Nieuwdorp M, Lok A, van Duijn CM, et al. Gut microbiome-wide association study of depressive symptoms. Nat Commun. 2022;13(1):7128. doi: 10.1038/s41467-022-34502-3. 36473852 PMC9726982

[36] Callahan BJ, Pj M, Rosen MJ, Han AW, Johnson AJ, Holmes SP. DADA2: high-resolution sample inference from illumina amplicon data. Nat Methods. 2016;13(7):581–583. doi: 10.1038/nmeth.3869. 27214047 PMC4927377

[37] Kim MS, Bae JW. Lysogeny is prevalent and widely distributed in the murine gut microbiota. SME J. 2018;12(4):1127–1141. doi: 10.1038/s41396-018-0061-9. PMC5864201 29416123

[38] Shin NR, Lee JC, Lee HY, Kim MS, Whon TW, Lee MS, Bae J-W. An increase in the Akkermansia spp. population induced by metformin treatment improves glucose homeostasis in diet-induced obese mice. Gut. 2014;63(5):727–735. doi: 10.1136/gutjnl-2012-303839. 23804561

[39] Segata N, Izard J, Waldron L, Gevers D, Miropolsky L, Garrett WS, Huttenhower C. Metagenomic biomarker discovery and explanation. Genome Biology. 2011;12(6):R60. doi: 10.1186/gb-2011-12-6-r60. 21702898 PMC3218848

[40] Derrien M, Vaughan EE, Plugge CM, de Vos WM. Akkermansia muciniphila gen. nov. sp. nov. a human intestinal mucin-degrading bacterium. Int J Syst Evol Microbiol. 2004;54(5):1469–1476. doi: 10.1099/ijs.0.02873-0. 15388697

[41] Hasani A, Ebrahimzadeh S, Hemmati F, Khabbaz A, Hasani A, Gholizadeh P. The role of Akkermansia muciniphila in obesity, diabetes and atherosclerosis. J Med Microbiol. 2021;70(10). doi: 10.1099/jmm.0.001435. 34623232

[42] Greer RL, Dong X, Moraes AC, Zielke RA, Fernandes GR, Peremyslova E, Vasquez-Perez S, Schoenborn AA, Gomes EP, Pereira AC, et al. Akkermansia muciniphila mediates negative effects of IFNγ on glucose metabolism. Nat Commun. 2016;7(1):13329. doi: 10.1038/ncomms13329. 27841267 PMC5114536

[43] Hernandez DJ, David AS, Menges ES, Searcy CA, Afkhami ME. Environmental stress destabilizes microbial networks. ISME J. 2021;15(6):1722–1734. doi: 10.1038/s41396-020-00882-x. 33452480 PMC8163744

[44] Masetti R, Leardini D, Muratore E, Fabbrini M, D’Amico F, Zama D, Baccelli F, Gottardi F, Belotti T, Ussowicz M, et al. Gut microbiota diversity before allogeneic hematopoietic stem cell transplantation as a predictor of mortality in children. Blood. 2023;142(16):1387–1398. doi: 10.1182/blood.2023020026. 37856089 PMC10651870

[45] Sun P, Wang M, Zheng W, Li S, Zhu X, Chai X, Zhao, S. Unbalanced diets enhance the complexity of gut microbial network but destabilize its stability and resistance. Stress Biol. 2023;3(1):20. doi: 10.1007/s44154-023-00098-x. 37676325 PMC10441997

[46] Chen P, Huang J, Rao L, Zhu W, Yu Y, Xiao F, Yu H, Wu Y, Hu R, Liu X, et al. Environmental effects of nanoparticles on the ecological succession of gut microbiota across zebrafish development. Sci Total Environ. 2022;806(Pt 4):150963. doi: 10.1016/j.scitotenv.2021.150963. 34656599

[47] Fabbrini M, D’Amico F, Leardini D, Muratore E, Barone M, Belotti T, Forchielli ML, Zama D, Pession A, Prete A, et al. Levofloxacin prophylaxis and parenteral nutrition have a detrimental effect on intestinal microbial networks in pediatric patients undergoing HSCT. Commun Biol. 2023;6(1):36. doi: 10.1038/s42003-023-04436-7. 36639555 PMC9839701

[48] Wilson LA, Sharp PM. Enterobacterial repetitive intergenic consensus (ERIC) sequences in Escherichia coli: evolution and implications for ERIC-PCR. Mol Biol Evol. 2006;23(6):1156–1168. doi: 10.1093/molbev/msj125. 16533821

[49] Choy CT, Wong CH, Chan SL. Embedding of genes using cancer gene expression data: biological relevance and potential application on biomarker discovery. Front Genet. 2018;9:682. doi: 10.3389/fgene.2018.00682. 30662451 PMC6329279

[50] Farin HF, Karthaus WR, Kujala P, Rakhshandehroo M, Schwank G, Vries RG, Kalkhoven E, Nieuwenhuis EES, Clevers H. Paneth cell extrusion and release of antimicrobial products is directly controlled by immune cell–derived IFN-γ. J Exp Med. 2014;211(7):1393–1405. doi: 10.1084/jem.20130753. 24980747 PMC4076587

[51] Chelakkot C, Choi Y, Kim DK, Park HT, Ghim J, Kwon Y, Jeon J, Kim M-S, Jee Y-K, Gho YS, et al. Akkermansia muciniphila-derived extracellular vesicles influence gut permeability through the regulation of tight junctions. Exp Mol Med. 2018;50(2):e450. doi: 10.1038/emm.2017.282. 29472701 PMC5903829

[52] Shi M, Yue Y, Ma C, Dong L, Chen F. Pasteurized Akkermansia muciniphila ameliorate the LPS-Induced intestinal barrier dysfunction via modulating AMPK and NF-κB through TLR2 in caco-2 cells. Nutrients. 2022;14(4):764. doi: 10.3390/nu14040764. 35215413 PMC8879293

[53] Zheng M, Han R, Yuan Y, Xing Y, Zhang W, Sun Z, Liu Y, Li J, Mao T. The role of Akkermansia muciniphila in inflammatory bowel disease: current knowledge and perspectives. Front Immunol. 2022;13:1089600. doi: 10.3389/fimmu.2022.1089600. 36685588 PMC9853388

[54] Blacher E, Bashiardes S, Shapiro H, Rothschild D, Mor U, Dori-Bachash M, Kleimeyer C, Moresi C, Harnik Y, Zur M, et al. Potential roles of gut microbiome and metabolites in modulating ALS in mice. Nature. 2019;572(7770):474–480. doi: 10.1038/s41586-019-1443-5. 31330533

[55] Li B, He Y, Ma J, Huang P, Du J, Cao L, Wang Y, Xiao Q, Tang H, Chen S. Mild cognitive impairment has similar alterations as Alzheimer’s disease in gut microbiota. Alzheimer's Dementia. 2019;15(10):1357–1366. doi: 10.1016/j.jalz.2019.07.002. 31434623

[56] Wang L, Christophersen CT, Sorich MJ, Gerber JP, Angley MT, Conlon MA. Low relative abundances of the mucolytic bacterium Akkermansia muciniphila and Bifidobacterium spp. In feces of children with autism. Appl Environ Microbiol. 2011;77(18):6718–6721. doi: 10.1128/AEM.05212-11. 21784919 PMC3187122

[57] McGaughey KD, Yilmaz-Swenson T, Elsayed NM, Cruz DA, Rodriguiz RM, Kritzer MD, Peterchev AV, Roach J, Wetsel WC, Williamson DE. Relative abundance of Akkermansia spp. And other bacterial phylotypes correlates with anxiety- and depressive-like behavior following social defeat in mice. Sci Rep. 2019;9(1):3281. doi: 10.1038/s41598-019-40140-5. 30824791 PMC6397238

[58] Sun Y, Zhu H, Cheng R, Tang Z, Zhang M. Outer membrane protein Amuc_1100 of Akkermansia muciniphila alleviates antibiotic-induced anxiety and depression-like behavior in mice. Physiol Behav. 2023;258:114023. doi: 10.1016/j.physbeh.2022.114023. 36336146

[59] Ding Y, Bu F, Chen T, Shi G, Yuan X, Feng Z, Duan Z, Wang R, Zhang S, Wang Q, et al. A next-generation probiotic: Akkermansia muciniphila ameliorates chronic stress–induced depressive-like behavior in mice by regulating gut microbiota and metabolites. Appl Microbiol Biotechnol. 2021;105(21–22):8411–8426. doi: 10.1007/s00253-021-11622-2. 34617139

[60] Guo H, Liu X, Chen T, Wang X, Zhang X. Akkermansia muciniphila improves depressive-like symptoms by modulating the level of 5-HT neurotransmitters in the gut and brain of mice. Mol Neurobiol. 2024;61(2):821–834. doi: 10.1007/s12035-023-03602-6. 37668965 PMC10861622

[61] Lei W, Cheng Y, Gao J, Liu X, Shao L, Kong Q, Zheng N, Ling Z, Hu W. Akkermansia muciniphila in neuropsychiatric disorders: friend or foe? Front Cell Infect Microbiol. 2023;13:1224155. doi: 10.3389/fcimb.2023.1224155. 37492530 PMC10363720

[62] Liu X, Cui Y, Zhang Y, Xiang G, Yu M, Wang X, Qiu B, Li X-G, Liu W, Zhang D. Rescue of social deficits by early-life melatonin supplementation through modulation of gut microbiota in a murine model of autism. Biomed Pharmacother. 2022;156:113949. doi: 10.1016/j.biopha.2022.113949. 36411634

[63] Kempuraj D, Thangavel R, Natteru PA, Selvakumar GP, Saeed D, Zahoor H, Zaheer S, Iyer SS, Zaheer A. Neuroinflammation induces Neurodegeneration. J Neurol Neurosurg Spine. 2016;1(1):1003. 28127589 PMC5260818

[64] Bian X, Wu W, Yang L, Lv L, Wang Q, Li Y, Ye J, Fang D, Wu J, Jiang X, et al. Administration of Akkermansia muciniphila ameliorates dextran sulfate sodium-induced ulcerative colitis in mice. Front Microbiol. 2019;10:2259. doi: 10.3389/fmicb.2019.02259. 31632373 PMC6779789

[65] Bae M, Cassilly CD, Liu X, Park SM, Tusi BK, Chen X, Kwon J, Filipčík P, Bolze AS, Liu Z, et al. Akkermansia muciniphila phospholipid induces homeostatic immune responses. Nature. 2022;608(7921):168–173. doi: 10.1038/s41586-022-04985-7. 35896748 PMC9328018

[66] Cheng R, Xu W, Wang J, Tang Z, Zhang M. The outer membrane protein Amuc_1100 of Akkermansia muciniphila alleviates the depression-like behavior of depressed mice induced by chronic stress. Biochem Biophys Res Commun. 2021;566:170–176. doi: 10.1016/j.bbrc.2021.06.018. 34129964

[67] Cani PD, de Vos WM. Next-generation beneficial microbes: the case of Akkermansia muciniphila. Front Microbiol. 2017;8:1765. doi: 10.3389/fmicb.2017.01765. 29018410 PMC5614963

[68] Di Filippo M, Mancini A, Bellingacci L, Gaetani L, Mazzocchetti P, Zelante T, La Barbera L, De Luca A, Tantucci M, Tozzi A, et al. Interleukin-17 affects synaptic plasticity and cognition in an experimental model of multiple sclerosis. Cell Rep. 2021;37(10):110094. doi: 10.1016/j.celrep.2021.110094. 34879272

[69] Mills KHG. IL-17 and IL-17-producing cells in protection versus pathology. Nat Rev Immunol. 2023;23(1):38–54. doi: 10.1038/s41577-022-00746-9. 35790881 PMC9255545

[70] Kang EJ, Cha MG, Kwon GH, Han SH, Yoon SJ, Lee SK, Ahn ME, Won S-M, Ahn EH, Suk KT. Akkermansia muciniphila improve cognitive dysfunction by regulating BDNF and serotonin pathway in gut-liver-brain axis. Microbiome. 2024;12(1):181. doi: 10.1186/s40168-024-01924-8. 39342324 PMC11438137

[71] Duman RS, Voleti B. Signaling pathways underlying the pathophysiology and treatment of depression: novel mechanisms for rapid-acting agents. Trends Neurosciences. 2012;35(1):47–56. doi: 10.1016/j.tins.2011.11.004. PMC3278537 22217452

[72] Kimono D, Bose D, Seth RK, Mondal A, Saha P, Janulewicz P, Sullivan K, Lasley S, Horner R, Klimas N, et al. Host akkermansia muciniphila abundance correlates with Gulf war illness symptom persistence via NLRP3-mediated Neuroinflammation and decreased brain-derived neurotrophic Factor. Neurosci Insights. 2020;15:2633105520942480. doi: 10.1177/2633105520942480. 32832901 PMC7440889

[73] Khaliulin I, Hamoudi W, Amal H. The multifaceted role of mitochondria in autism spectrum disorder. Mol Psychiatry. 2025;30(2):629–650. doi: 10.1038/s41380-024-02725-z. 39223276 PMC11753362

[74] Gimenez-Palomo A, Dodd S, Anmella G, Carvalho AF, Scaini G, Quevedo J, Pacchiarotti I, Vieta E, Berk M. The role of mitochondria in mood disorders: from physiology to pathophysiology and to treatment. Front Psychiatry. 2021;12:546801. doi: 10.3389/fpsyt.2021.546801. 34295268 PMC8291901

[75] Li Z, Okamoto K, Hayashi Y, Sheng M. The importance of dendritic mitochondria in the morphogenesis and plasticity of spines and synapses. Cell. 2004;119(6):873–887. doi: 10.1016/j.cell.2004.11.003. 15607982

[76] Kimura T, Murakami F. Evidence that dendritic mitochondria negatively regulate dendritic branching in pyramidal neurons in the neocortex. J Neurosci. 2014;34(20):6938–6951. doi: 10.1523/JNEUROSCI.5095-13.2014. 24828647 PMC6608104

[77] Daniels TE, Olsen EM, Tyrka AR. Stress and psychiatric disorders: the role of mitochondria. Annu Rev Clin Psychol. 2020;16(1):165–186. doi: 10.1146/annurev-clinpsy-082719-104030. 32092280 PMC8007172

[78] Al-Kafaji G, Sabry MA, Bakhiet M. Increased expression of mitochondrial DNA-encoded genes in human renal mesangial cells in response to high glucose-induced reactive oxygen species. Mol Med Rep. 2016;13(2):1774–1780. doi: 10.3892/mmr.2015.4732. 26719045

[79] Kaare M, Mikheim K, Lillevali K, Kilk K, Jagomae T, Leidmaa E, Piirsalu M, Porosk R, Singh K, Reimets R, et al. High-fat diet induces pre-diabetes and distinct Sex-specific metabolic alterations in Negr1-deficient mice. Biomedicines. 2021;9(9):1148. doi: 10.3390/biomedicines9091148. 34572334 PMC8466019

[80] Wang Z, Wang C, Yuan B, Liu L, Zhang H, Zhu M, Chai H, Peng J, Huang Y, Zhou S, et al. Akkermansia muciniphila and its metabolite propionic acid maintains neuronal mitochondrial division and autophagy homeostasis during Alzheimer’s disease pathologic process via GPR41 and GPR43. Microbiome. 2025;13(1):16. doi: 10.1186/s40168-024-02001-w. 39833898 PMC11744907

[81] Zhai R, Xue X, Zhang L, Yang X, Zhao L, Zhang C. Strain-specific anti-inflammatory properties of two Akkermansia muciniphila strains on chronic colitis in mice. Front Cell Infect Microbiol. 2019;9:239. doi: 10.3389/fcimb.2019.00239. 31334133 PMC6624636

[82] Liu Y, Yang M, Tang L, Wang F, Huang S, Liu S, Lei Y, Wang S, Xie Z, Wang W, et al. TLR4 regulates RORγt+ regulatory T-cell responses and susceptibility to colon inflammation through interaction with Akkermansia muciniphila. Microbiome. 2022;10(1):98. doi: 10.1186/s40168-022-01296-x. 35761415 PMC9235089

[83] Wang L, Tang L, Feng Y, Zhao S, Han M, Zhang C, Yuan G, Zhu J, Cao S, Wu Q, et al. A purified membrane protein from Akkermansia muciniphila or the pasteurised bacterium blunts colitis associated tumourigenesis by modulation of CD8 + T cells in mice. Gut. 2020;69(11):1988–1997. doi: 10.1136/gutjnl-2019-320105. 32169907 PMC7569398

[84] Qian Y, Kang Z, Liu C, Li X. IL-17 signaling in host defense and inflammatory diseases. Cell Mol Immunol. 2010;7(5):328–333. doi: 10.1038/cmi.2010.27. 20514051 PMC4002676

[85] Zenobia C, Hajishengallis G. Basic biology and role of interleukin-17 in immunity and inflammation. Periodontol. 2000;69(1):142–159. doi: 10.1111/prd.12083. PMC4530463 26252407

[86] Davami MH, Baharlou R, Ahmadi Vasmehjani A, Ghanizadeh A, Keshtkar M, Dezhkam I, Atashzar M. Elevated IL-17 and TGF-β serum levels: a positive correlation between T-helper 17 cell-related pro-inflammatory responses with major depressive disorder. Basic Clin Neurosci. 2016;7(2):137–142. doi: 10.15412/J.BCN.03070207. 27303608 PMC4892318

[87] Blizniewska-Kowalska K, Szewczyk B, Galecka M, Su KP, Maes M, Szemraj J, Gałecki P. Is interleukin 17 (IL-17) expression a common point in the pathogenesis of depression and obesity? J Clin Med. 2020;9(12):4018. doi: 10.3390/jcm9124018. 33322667 PMC7763002

[88] Galecka M, Blizniewska-Kowalska K, Orzechowska A, Szemraj J, Maes M, Berk M, Su K-P, Gałecki P. Inflammatory versus anti-inflammatory profiles in Major depressive disorders—The role of IL-17, IL-21, IL-23, IL-35 and Foxp3. J Personalized Med. 2021;11(2):66. doi: 10.3390/jpm11020066. PMC7911855 33498653

[89] Kim J, Suh YH, Chang KA. Interleukin-17 induced by cumulative mild stress promoted depression-like behaviors in young adult mice. Mol Brain. 2021;14(1):11. doi: 10.1186/s13041-020-00726-x. 33441182 PMC7805143

[90] Reed MD, Yim YS, Wimmer RD, Kim H, Ryu C, Welch GM, Andina M, King HO, Waisman A, Halassa MM, et al. IL-17a promotes sociability in mouse models of neurodevelopmental disorders. Nature. 2020;577(7789):249–253. doi: 10.1038/s41586-019-1843-6. 31853066 PMC8112727

[91] Leonardi I, Gao IH, Lin WY, Allen M, Li XV, Fiers WD, De Celie MB, Putzel GG, Yantiss RK, Johncilla M, et al. Mucosal fungi promote gut barrier function and social behavior via Type 17 immunity. Cell. 2022;185(5):831–846.e14. doi: 10.1016/j.cell.2022.01.017. 35176228 PMC8897247

[92] Plovier H, Everard A, Druart C, Depommier C, Van Hul M, Geurts L, Chilloux J, Ottman N, Duparc T, Lichtenstein L, et al. A purified membrane protein from Akkermansia muciniphila or the pasteurized bacterium improves metabolism in obese and diabetic mice. Nat Med. 2017;23(1):107–113. doi: 10.1038/nm.4236. 27892954

[93] Costea PI, Zeller G, Sunagawa S, Pelletier E, Alberti A, Levenez F, Tramontano M, Driessen M, Hercog R, Jung F-E, et al. Towards standards for human fecal sample processing in metagenomic studies. Nat Biotechnol. 2017;35(11):1069–1076. doi: 10.1038/nbt.3960. 28967887

[94] Zoetendal EG, Heilig HG, Klaassens ES, Booijink CC, Kleerebezem M, Smidt H, de Vos WM. Isolation of DNA from bacterial samples of the human gastrointestinal tract. Nat Protoc. 2006;1(2):870–873. doi: 10.1038/nprot.2006.142. 17406319

[95] Kim MS, Park EJ. Postharvest-induced microbiota remodeling increases fungal diversity in the phyllosphere mycobiota of broccoli florets. Postharvest Biol Tec. 2021;181:111693. doi: 10.1016/j.postharvbio.2021.111693.

[96] Schwager E, Bielski C, Weingart G. ccrepe: ccrepe_and_nc.score. R package version 1.24.0. 2020. doi: 10.1038/s41564-018-0202-y.

[97] Clauset A, Newman ME, Moore C. Finding community structure in very large networks. Phys Rev E Stat Nonlin Soft Matter Phys. 2004;70(6 Pt 2):066111. doi: 10.1103/PhysRevE.70.066111. 15697438

[98] Guo X, Zhang J, Wu F, Zhang M, Yi M, Peng Y. Different subtype strains of Akkermansia muciniphila abundantly colonize in southern China. J Appl Microbiol. 2016;120(2):452–459. doi: 10.1111/jam.13022. 26666632 PMC4736461

[99] Zaqout S, Kaindl AM. Golgi-cox staining step by step. Front Neuroanat. 2016;10:38. doi: 10.3389/fnana.2016.00038. 27065817 PMC4814522

[100] Lee CY, Hyun SA, Ko MY, Kim HR, Rho J, Kim KK, Kim W-Y, Ka M. Maternal bisphenol a (BPA) exposure alters cerebral cortical morphogenesis and synaptic function in mice. Cereb Cortex. 2021;31(12):5598–5612. doi: 10.1093/cercor/bhab183. 34171088

[101] Walf AA, Frye CA. The use of the elevated plus maze as an assay of anxiety-related behavior in rodents. Nat Protoc. 2007;2(2):322–328. doi: 10.1038/nprot.2007.44. 17406592 PMC3623971

[102] Can A, Dao DT, Arad M, Terrillion CE, Piantadosi SC, Gould TD. The mouse forced swim test. J Visualized Experiments: JoVE. 2012 59. (59):e3638. doi: 10.3791/3638-v. PMC3353513 22314943

[103] Love MI, Huber W, Anders S. Moderated estimation of Fold change and dispersion for RNA-seq data with DESeq2. Genome Biol. 2014;15(12):550. doi: 10.1186/s13059-014-0550-8. 25516281 PMC4302049

[104] Muzellec B, Telenczuk M, Cabeli V, Andreux M, Ponty Y. PyDESeq2: a python package for bulk RNA-seq differential expression analysis. Bioinformatics. 2023;39(9). doi: 10.1093/bioinformatics/btad547. PMC10502239 37669147

[105] Kuleshov MV, Jones MR, Rouillard AD, Fernandez NF, Duan Q, Wang Z, Koplev S, Jenkins SL, Jagodnik KM, Lachmann A, et al. Enrichr: a comprehensive gene set enrichment analysis web server 2016 update. Nucleic Acids Res. 2016;44(W1):W90–7. doi: 10.1093/nar/gkw377. 27141961 PMC4987924

[106] Dixon P. VEGAN, a package of R functions for community ecology. J Veg Sci. 2003;14(6):927–930. doi: 10.1111/j.1654-1103.2003.tb02228.x.

