# Supplementary material for: Akkermansia muciniphila reverses neuronal atrophy in Negr1 knockout mice with depression-like phenotypes
Source: Gut Microbes. 2025 May 19;17(1):2508424. doi: 10.1080/19490976.2025.2508424 (PMC12091914; doi:10.1080/19490976.2025.2508424)
Supplement: Suppl_Figures_Revision_JYKIM.docx [file KGMI_A_2508424_SM4878.docx]

**A**


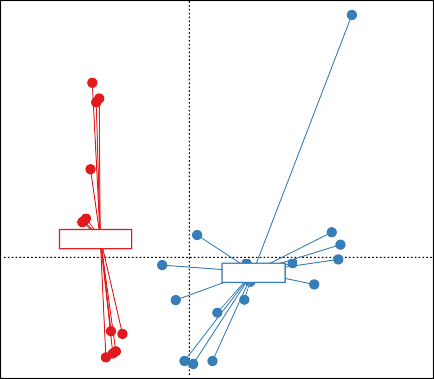


6-wk (*P* = 0.001)

*Negr1* KO

WT

PCo1 (24.9%)


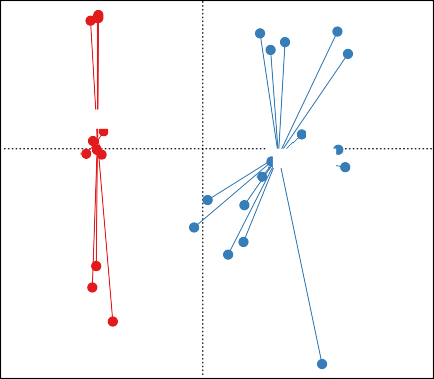


9-wk (*P* = 0.001)

PCo1 (22.1%)

WT

*Negr1* KO

PCo2 (16.5%)

PCo2 (12.7%)

**B** 400


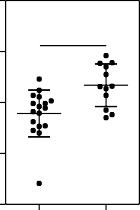


*P* = 0.004

300

The number of ASVs

200

100

6

5


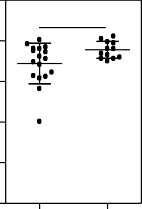


1.0

*P* = 0.054

0.8

0.6

0.4

0.2

0.0


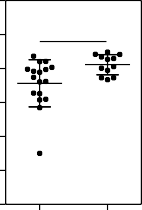


*P* = 0.0096

4

Shannon index

3

2

1

400

300


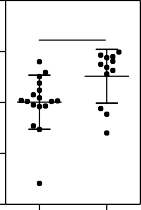


*P* = 0.0201

The number of ASVs

200

100

1.0 6

0.8 5


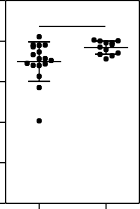


*P* = 0.0109


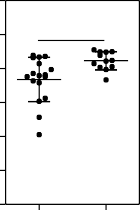


*P* = 0.0057

Pielou's evenness

4

Shannon index

0.6

3

0.4

2

0.2 1

0

Pielou's evenness

WT Negr1 KO

W Tr1

0

WT Negr1 KO

0

WT Negr1 KO

0.0

WT Negr1 KO

0

WT Negr1 KO

### 9-wk 6-wk


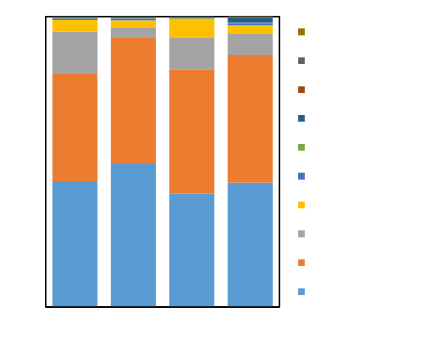


**C** 100%

90%

Unclassified

Cyanobacteria

80%

Actinobacteria

70%

60%

50%

TM7

Deferribacteres Tenericutes

40%

Proteobacteria

30%

Verrucomicrobia

20%

Firmicutes

10%

Bacteroidetes

0%

WT *Negr1* KO WT *Negr1* KO


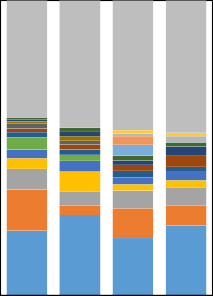
100%
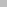
 Others

90%

80%

70%

60%

50%

40%

30%

20%

10%

0%

WT *Negr1* KO WT *Negr1* KO


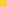
Clostridiaceae;Clostridium


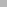
Flexispira
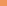
 Sulcia
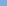
 Allobaculum
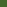
 Coprococcus
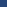
 Paraprevotella
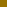
 Sutterella
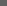
 Parabacteroides
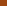
 Lactobacillus


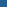
Lachnospiraceae;Ruminococcus
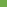
 Blautia


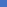
Oscillospira
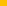
 Bacteroides
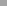
 Prevotella
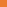
 Akkermansia
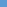
 S24-7

**D**

Firmicutes; Lachnospiraceae Bacteroidetes; Bacteroides Firmicutes; Clostridiales Firmicutes; Lachnospiraceae Bacteroidetes; S24-7 Firmicutes; Lachnospiraceae Firmicutes; Clostridiales Bacteroidetes; S24-7 Bacteroidetes; S24-7 Firmicutes; Lachnospiraceae Firmicutes; Lachnospiraceae Bacteroidetes; S24-7 Bacteroidetes; S24-7 Firmicutes; Lachnospiraceae Bacteroidetes; S24-7 Firmicutes; Lachnospiraceae Firmicutes; Lachnospiraceae Firmicutes; Ruminococcus Firmicutes; Lachnospiraceae Firmicutes; Ruminococcus Firmicutes; Butyricicoccus Firmicutes; Coprococcus Firmicutes; Clostridiales Firmicutes; Lachnospiraceae Firmicutes; Lachnospiraceae Bacteroidetes; S24-7 Firmicutes; Lachnospiraceae Firmicutes; Ruminococcus Firmicutes; Coprococcus Bacteroidetes; S24-7 Firmicutes; Lachnospiraceae


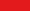
 *Negr1* KO
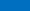
 WT **E**


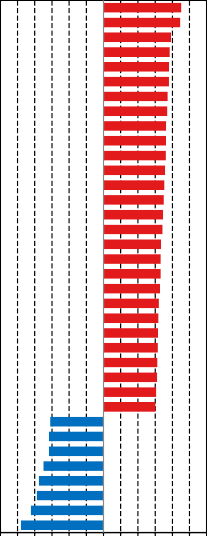

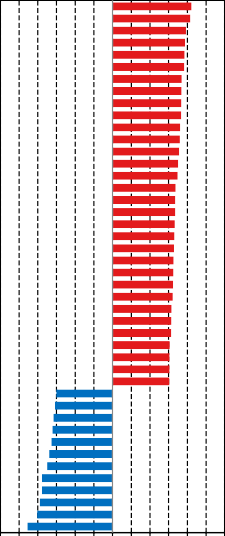
Firmicutes; Lachnospiraceae Firmicutes; Clostridiales Firmicutes; Lachnospiraceae

TM7

Firmicutes; Lachnospiraceae Bacteroidetes; S24-7 Firmicutes; Clostridiaceae Bacteroidetes; Rikenellaceae Firmicutes; Lachnospiraceae Firmicutes; Lachnospiraceae Firmicutes; Clostridiales Firmicutes; Clostridiales Bacteroidetes; S24-7 Firmicutes; Lachnospiraceae Firmicutes; Lachnospiraceae Firmicutes; Lachnospiraceae Firmicutes; Lachnospiraceae Firmicutes; Ruminococcus Bacteroidetes; S24-7 Bacteroidetes; S24-7 Firmicutes; Coprococcus Bacteroidetes; S24-7 Firmicutes; Ruminococcus Firmicutes; Lachnospiraceae Firmicutes; Clostridiales

TM7

Firmicutes; Coprococcus Firmicutes; Ruminococcus Firmicutes; Clostridiales Firmicutes; Clostridiales Firmicutes; Lachnospiraceae Bacteroidetes; S24-7 Firmicutes; Lachnospiraceae Firmicutes; Clostridiales Firmicutes; Ruminococcus Bacteroidetes; Parabacteroides Firmicutes; Lachnospiraceae Proteobacteria; Desulfovibrio

50

40


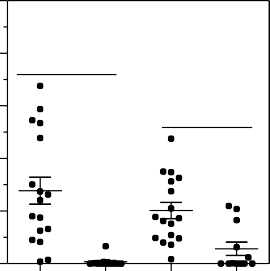


*P* < 0.0001

*P* = 0.0018

Relative abundance (%)

30

20

10

0

WT Negr1 KO WT Negr1 KO

#### 6-wk 9-wk

Bacteroidetes; Parabacteroides Proteobacteria; Helicobacter Firmicutes; Clostridiales Bacteroidetes; Prevotella Verrucomicrobia; Akkermansia

-6 -5 -4 -3 -2 -1 0 1 2 3 4 5 6

LDA score (Log10)

Firmicutes; Ruminococcus Proteobacteria; Helicobacter Firmicutes; Allobaculum Firmicutes; Allobaculum Firmicutes; Clostridiales Verrucomicrobia; Akkermansia

-6 -5 -4 -3 -2 -1 0 1 2 3 4 5 6

LDA score (Log10)

**Supplementary Fig. 1**

**A**

**B**


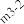


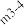


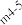


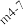


**
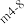
**


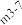


**
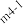
**

Identity (%)

**C**


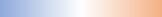


94 100


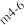

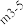

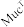


m3-2 m3-4 m3-6

m4-5 m4-7 m4-8

m3-7 m4-1

m4-6 m3-5

MucT PytT

Identity (%)


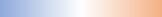


*Akkermansia glycaniphila* PytT m4-5

#### m4-7 m3-6 m3-4 m4-8

*A. muciniphila* MucT

*A. muciniphila* YL44

*A. muciniphila* WW001

*A. muciniphila* CSUN-33

*A. muciniphila* 2SK-D10-15

84 *A. muciniphila* MGYG-HGUT-02454

*A. muciniphila* GP28

*A. muciniphila* EB-AMDK-36

*A. muciniphila* TL23 m4-6

*Akkermansia* ASV1 *Akkermansia* ASV4 m3-7


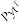

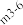


100 99.85 99.85 99.85 99.85 99.85 99.85 99.85

99.46 99.46

99.85 99.92 99.92 99.92 99.92 99.92 100 100 99.85

99.85 99.92 99.92 99.92 99.92 99.92 100 100 99.85

99.92 99.92 99.92 99.92 99.92 99.85 99.85 100

94 94.14 94.14 94.14 94.14 94.14 94.07 94.07 94.07 93.99 93.91 100

100

99.46 99.61 99.61 99.61 99.61 99.61 99.54 99.54 99.54 99.46

100

99.77 99.77 99.77

99.69 99.85 99.85 99.85 99.85 99.85

99.77

99.77

99.77

99.77

99.61

99.61

99.61

99.61

99.61

99.56

99.56

99.56

99.46

99.85 100 100 100 100 100 99.92 99.92 99.92 99.85

99.85 100 100 100 100 100 99.92 99.92 99.92 99.85

99.85 100 100 100 100 100 99.92 99.92 99.92 99.85

99.85 100 100 100 100 100 99.92 99.92 99.92 99.85

99.85 100 100 100 100 100 99.92 99.92 99.92 99.85

94

94.14

94.14

94.14

94.14

94.14

94.07

94.07

94.07

93.99

93.91

99.77

89 100


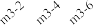

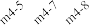


#### ^91^ m4-1 m3-2

*Akkermansia* ASV2 *Akkermansia* ASV3 m3-5

*A. muciniphila* m1-15

##### A. muciniphila H2

*A. muciniphila* Akk0500b

*A. muciniphila* KGMB01990

*A. muciniphila* AMUC

100 *A. muciniphila* JCM30893


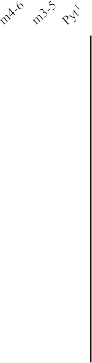

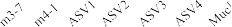


| 100 | 100 | 100 | 100 | 100 | 100 | 100 | 100 | 100 | 100 | 100 | 100 | 100 99.62 99.23 90.39 m3-2 |
| --- | --- | --- | --- | --- | --- | --- | --- | --- | --- | --- | --- | --- |
| 100 | 100 | 100 | 100 | 100 | 100 | 100 | 100 | 100 | 100 | 100 | 100 | 100 99.62 99.23 90.39 m3-4 |
| 100 | 100 | 100 | 100 | 100 | 100 | 100 | 100 | 100 | 100 | 100 | 100 | 100 99.62 99.23 90.39 m3-6 |
| 100 | 100 | 100 | 100 | 100 | 100 | 100 | 100 | 100 | 100 | 100 | 100 | 100 99.62 99.23 90.39 m4-5 |
| 100 | 100 | 100 | 100 | 100 | 100 | 100 | 100 | 100 | 100 | 100 | 100 | 100 99.62 99.23 90.39 m4-7 |
| 100 | 100 | 100 | 100 | 100 | 100 | 100 | 100 | 100 | 100 | 100 | 100 | 100 99.62 99.23 90.39 m4-8 |
| 100 | 100 | 100 | 100 | 100 | 100 | 100 | 100 | 100 | 100 | 100 | 100 | 100 99.62 99.23 90.39 m3-7 |
| 100 | 100 | 100 | 100 | 100 | 100 | 100 | 100 | 100 | 100 | 100 | 100 | 100 99.62 99.23 90.39 m4-1 |
| 100 | 100 | 100 | 100 | 100 | 100 | 100 | 100 | 100 | 100 | 100 | 100 | 100 99.62 99.23 90.39 ASV1 |
| 100 | 100 | 100 | 100 | 100 | 100 | 100 | 100 | 100 | 100 | 100 | 100 | 100 99.62 99.23 90.39 ASV2 |
| 100 | 100 | 100 | 100 | 100 | 100 | 100 | 100 | 100 | 100 | 100 | 100 | 100 99.62 99.23 90.39 ASV3 |
| 100 | 100 | 100 | 100 | 100 | 100 | 100 | 100 | 100 | 100 | 100 | 100 | 100 99.62 99.23 90.39 ASV4 |
| 100 | 100 | 100 | 100 | 100 | 100 | 100 | 100 | 100 | 100 | 100 | 100 | 100 99.62 99.23 90.39 MucT |
| 99.62 99.62 99.62 99.62 99.62 99.62 99.62 99.62 99.62 99.62 99.62 99.62 99.62 100 98.86 90.04 m4-6  99.23 99.23 99.23 99.23 99.23 99.23 99.23 99.23 99.23 99.23 99.23 99.23 99.23 98.86 100 89.66 m3-5  90.39 90.39 90.39 90.39 90.39 90.39 90.39 90.39 90.39 90.39 90.39 90.39 90.39 90.04 90.39 100 Pyt^T^ | | | | | | | | | | | | |

*A. muciniphila* CBA5201

0.10 *A. muciniphila* 139

**D**


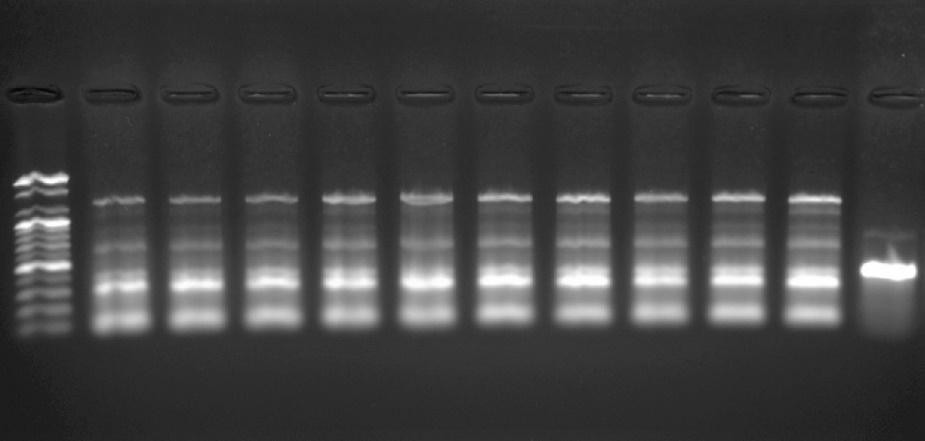


**m3-2**

**m3-4**

**m3-5**

**m3-6**

**m3-7**

**m4-1**

**m4-5**

**m4-6**

**m4-7**

**m4-8 MucT**

**2,000 bp**

**1,000 bp**

**500 bp**

**100 bp**

**Supplementary Fig. 2**

**A** Birth 7-wk


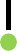


11-wk

15-wk


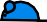
 WT + PBS WT + AKK


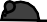

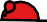

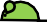


*Negr1* KO + PBS

*Negr1* KO + AKK

##### Akkermansia spp. m3-2 or PBS treatment

*Behavioral tests*

- fecal samples
- brain samples

11.0 10.0

*P* = 0.6200

The number of CFUs (log)

**B**

**C**

10.5

The number of CFUs (log)

10.0

9.5

9.5 9.0

9.0

8.5

8.5

8.0

m3-2 suspension

8.0

Before After

**Supplementary Fig. 3**

**
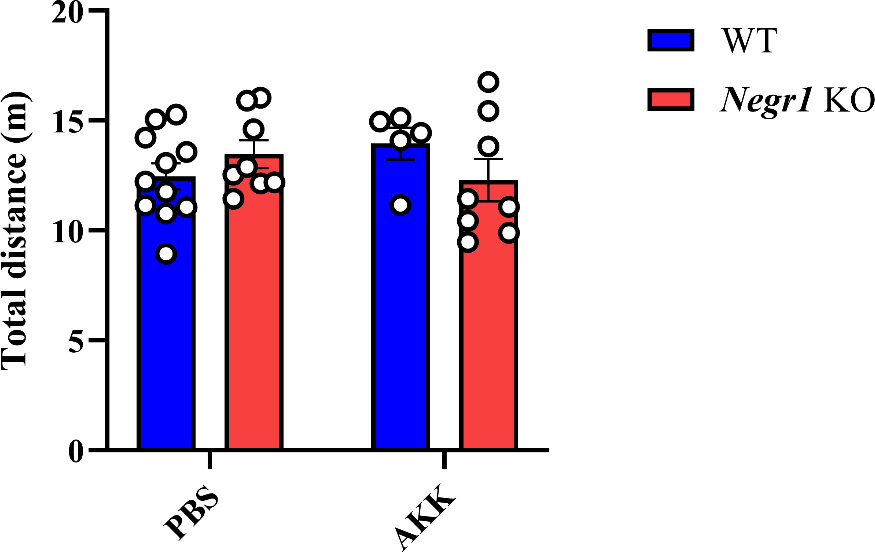
****A**

**
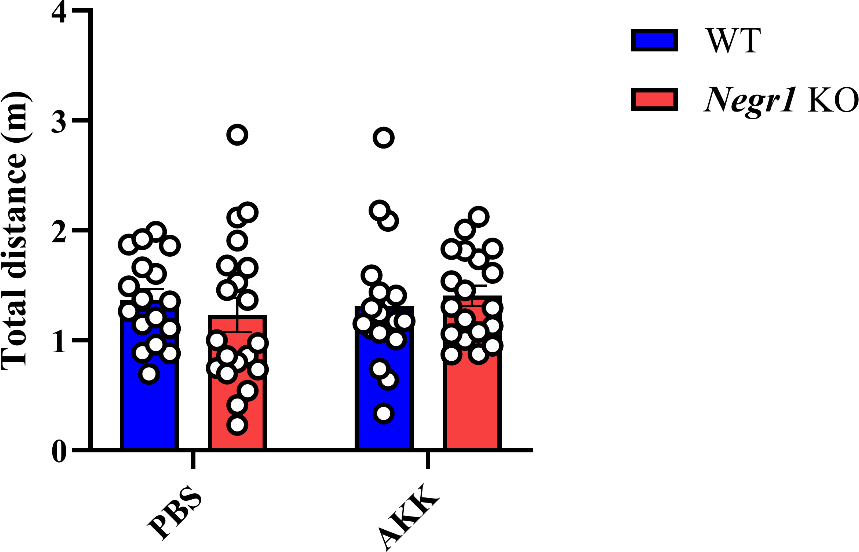
B**

**
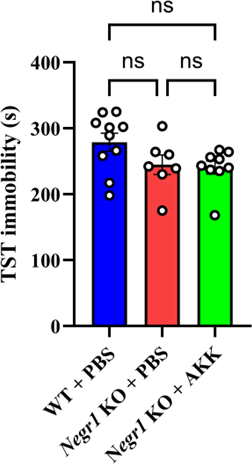

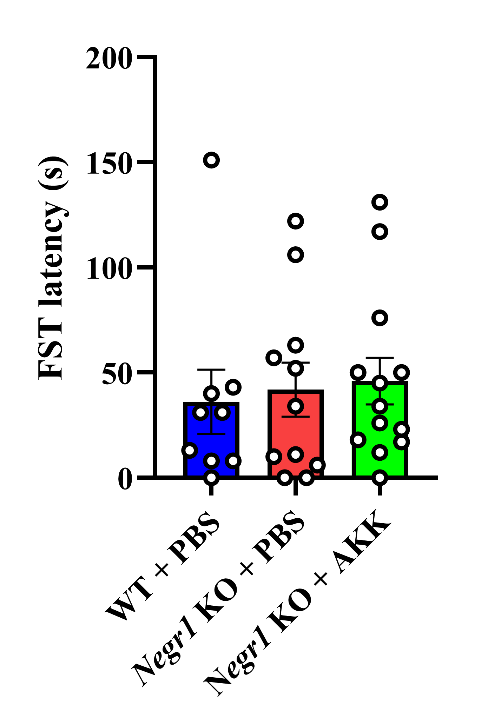
C**

**D**

**Supplementary Fig. 4**

**
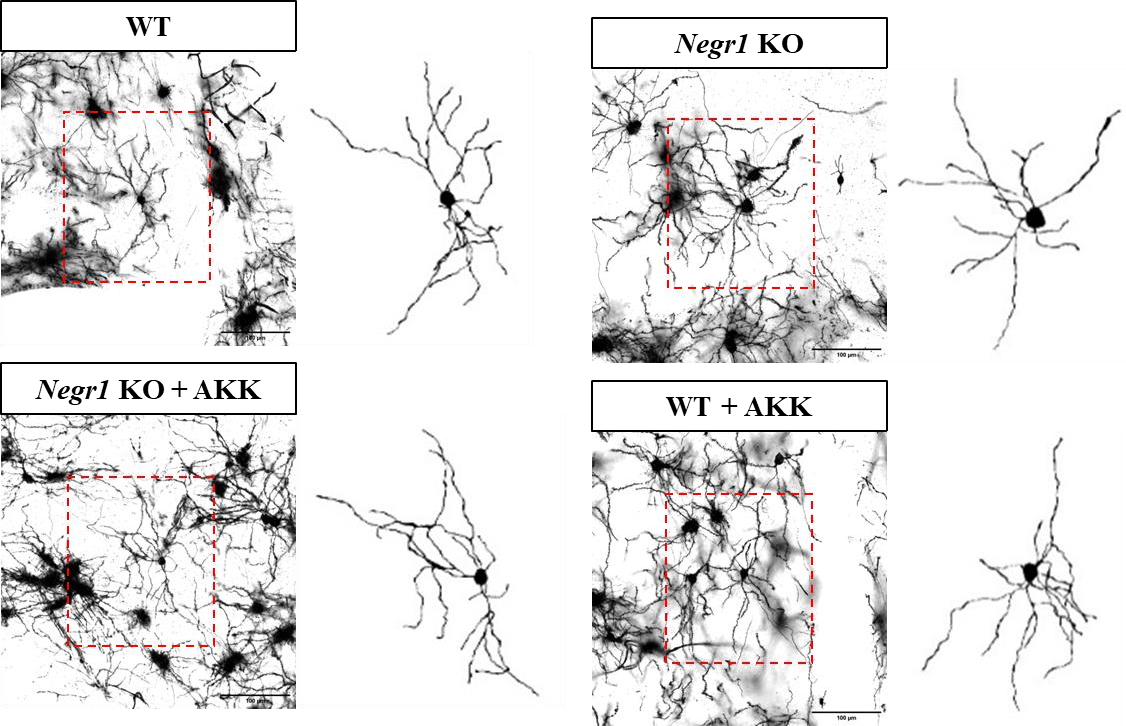
****A**

**
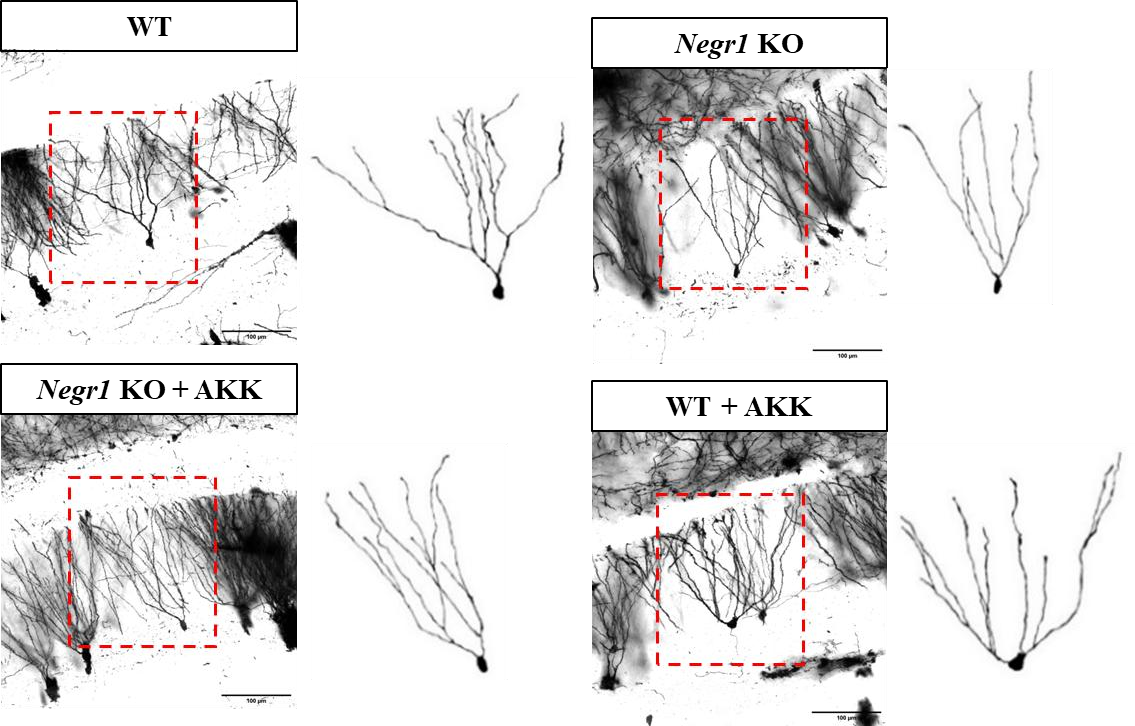
B**

**Supplementary Fig. 5**

**A B**


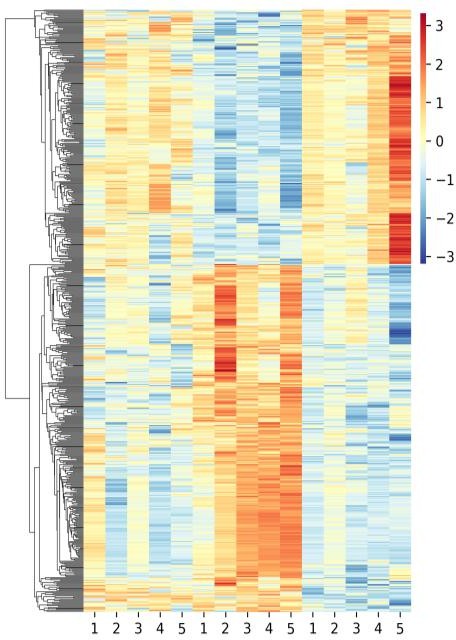

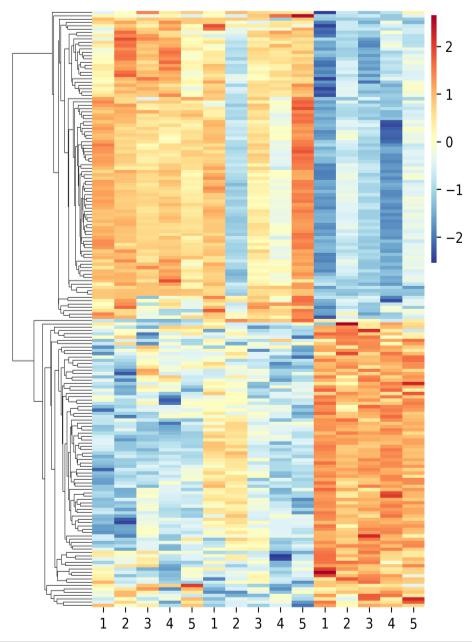


Log2(Fold change)

Log2(Fold change)

#### WT Negr1 KO Negr1 KO

+ AKK

1. Aminoacyl-tRNA biosynthesis Oxidative phosphorylation

Diabetic cardiomyopathy Retrograde endocannabinoid signaling

Thermogenesis Parkinson disease

Neuroactive ligand-receptor interaction

#### WT Negr1 KO Negr1 KO

+ AKK

1. NOD-like receptor signaling pathway Hepatitis C Influenza A

Measles Epstein-Barr virus infection Coronavirus disease

WT+PBS KO+PBS KO+AKK

Prion disease Huntington disease Alzheimer disease Osteoclast differentiation

Parathyroid hormone synthesis secretion and action C-type lectin receptor signaling pathway

IL-17 signaling pathway

Kaposi sarcoma-associated herpesvirus infection

Necroptosis Herpes simplex virus 1 infection Staphylococcus aureus infection Renin-angiotensin system

-4 -2 0 2 4

-4 -2 0 2 4

Enrichment log(combined score)

Circadian entrainment Insulin secretion

ECM-receptor interaction Calcium signaling pathway Apelin signaling pathway

Adrenergic signaling in cardiomyocytes

Ribosome Huntington disease Prion disease

Neuroactive ligand-receptor interaction

Parkinson disease Retrograde endocannabinoid signaling

Thermogenesis Diabetic cardiomyopathy Oxidative phosphorylation Aminoacyl-tRNA biosynthesis

-4 -2 0 2 4

Enrichment log(combined score)

Enrichment log(combined score)

T cell receptor signaling pathway Primary immunodeficiency

PD-L1 expression and PD-1 checkpoint pathway in cancer

Th1 and Th2 cell differentiation Natural killer cell mediated cytotoxicity

Th17 cell differentiation Cell adhesion molecules

Yersinia infection Fc epsilon RI signaling pathway Non-small cell lung cancer Diabetic cardiomyopathy

Retrograde endocannabinoid signaling

Huntington disease Coronavirus disease

Prion disease Parkinson disease Oxidative phosphorylation

Non-alcoholic fatty liver disease

Ribosome Sulfur metabolism

-4 -2 0 2 4

Enrichment log(combined score)

Primary immunodeficiency Maturity onset diabetes of the young T cell receptor signaling pathway

Viral protein interaction with cytokine and cytokine receptor

Chemokine signaling pathway Th1 and Th2 cell differentiation Th17 cell differentiation

PD-L1 expression and PD-1 checkpoint pathway in cancer

Measles Natural killer cell mediated cytotoxicity

Diabetic cardiomyopathy Huntington disease Prion disease

Non-alcoholic fatty liver disease

Thermogenesis Coronavirus disease Sulfur metabolism Parkinson disease Oxidative phosphorylation

Ribosome

-3 -2 -1 0 1 2 3

Enrichment log(combined score)

**Supplementary Figure 6.**

1. **OXPHOS (mtDNA)**

**
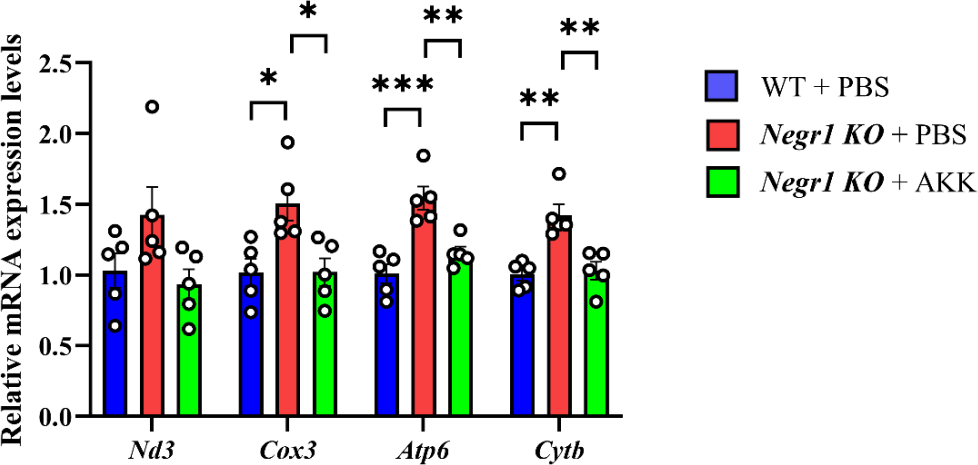
**

1. **IL17 signaling pathway**

**
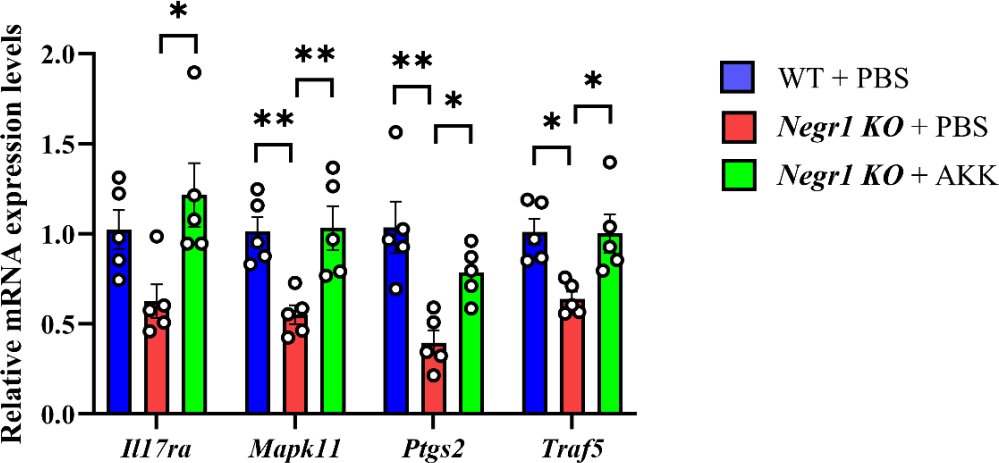
**

**Supplementary Fig. 7**

**A** 35 10 25


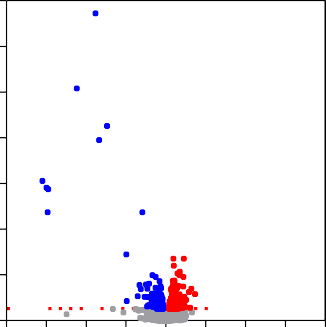


WT+PBS (139)

KO+PBS (299)


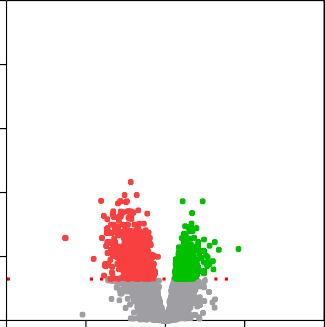


KO+PBS (632)

KO+AKK (436)


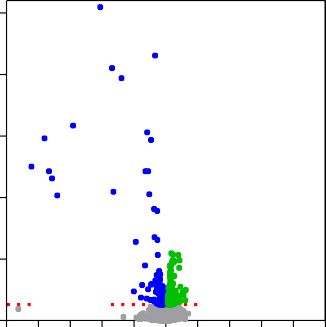


WT+PBS (115)

KO+AKK (236)

30

8

20

25

-Log_10_(adj.*P*-value)

-Log_10_(adj.*P*-value)

-Log_10_(adj.*P*-value)

20 6 15

15 4 10

10

2 5

5

0

-8 -6 -4 -2 0 2 4 6 8

Log_2_ Fold Change

# B

0

-2 -1 0 1 2

###### Log_2_ Fold Change

0

-10 -8 -6 -4 -2 0 2 4 6 8 10

###### Log_2_ Fold Change

WT+PBS

KO+PBS

KO+AKK

Mitochondrial Protein Catabolic Process Cellular Response To Histamine Pharyngeal Arch Artery Morphogenesis

Mitral Valve Development Inhibitory Synapse Assembly Synaptic Transmission GABAergic Glucan Metabolic Process

Negative Regulation Of Ubiquitin Protein Ligase Activity UDP-N-acetylglucosamine Biosynthetic Process Regulation Of Humoral Immune Response Regulation Of Fatty Acid Beta-Oxidation

Positive Regulation Of T-helper 1 Type Immune Response Cellular Response To Leucine Starvation Regulation Of mRNA Processing

Response To Leucine Cellular Response To Leucine Translational Termination

Regulation Of mRNA Metabolic Process Diacylglycerol Biosynthetic Process Regulation Of Protein Glycosylation

-4 -2 0 2 4

Protein Localization To Endoplasmic Reticulum Exit Site Regulation Of Wound Healing Spreading Of Epidermal Cells

Protein Localization To Golgi Apparatus Negative Regulation Of Neuron Migration Negative Regulation Of Cellular Extravasation

Synaptic Vesicle Uncoating Response To DNA Damage Checkpoint Signaling Regulation Of Ribonucleoprotein Complex Localization Regulation Of mRNA Export From Nucleus

Positive Regulation Of Transcription By RNA Polymerase I Mitochondrial Electron Transport Ubiquinol To Cytochrome C

Aerobic Respiration Mitochondrial Electron Transport NADH To Ubiquinone

Translation Macromolecule Biosynthetic Process Peptide Biosynthetic Process

Mitochondrial ATP Synthesis Coupled Electron Transport

Cellular Respiration Aerobic Electron Transport Chain Cytoplasmic Translation

-4 -2 0 2 4

Presynaptic Active Zone Organization

Imitative Learning Vocal Learning

Tight Junction Organization Chondrocyte Development

Positive Regulation Of Amyloid-Beta Clearance

Pituitary Gland Development Release Of Sequestered Ca2+ Into Cytosol By SR

Dendritic Spine Maintenance Release Of Sequestered Ca2+ Into Cytosol By ER Proton Motive Force-Driven ATP Synthesis Macromolecule Biosynthetic Process

Proton Motive Force-Driven Mitochondrial ATP Synthesis Mitochondrial ATP Synthesis Coupled Electron Transport

Peptide Biosynthetic Process Aerobic Electron Transport Chain

Translation Oxidative Phosphorylation Aerobic Respiration Cytoplasmic Translation

-4 -2 0 2 4

Enrichment log(combined score)

**C**

Enrichment log(combined score)

Enrichment log(combined score)

Bone Morphogenic Protein Signaling And Regulation

Oxidative Phosphorylation Mitochondrial Complex I Assembly Model OXPHOS System Electron Transport Chain OXPHOS System In Mitochondria mBDNF And proBDNF Regulation Of GABA Neurotransmission

P53 Signaling

-4 -2 0 2 4

mRNA Processing Oxidative Stress And Redox Pathway Selenium Micronutrient Network

mRNA Processing Eukaryotic Transcription Initiation

TCA Cycle Proteasome Degradation Oxidative Phosphorylation Electron Transport Chain Cytoplasmic Ribosomal Proteins

Dravet Syndrome Scn1a A1783V Point Mutation Model

ID Signaling Pathway Mechanisms Associated With Pluripotency

SIDS Susceptibility Pathways Wnt Signaling Pathway

Calcium Regulation In Cardiac Cells

P53 Signaling Oxidative Phosphorylation

TCA Cycle Electron Transport Chain Cytoplasmic Ribosomal Proteins

Enrichment log(combined score)

**D**

Aminoacyl-tRNA biosynthesis Retrograde endocannabinoid signaling

Oxidative phosphorylation TGF-beta signaling pathway

Parkinson disease Diabetic cardiomyopathy Amyotrophic lateral sclerosis

Prion disease Huntington disease Thermogenesis Insulin resistance

Longevity regulating pathway Small cell lung cancer p53 signaling pathway

mRNA surveillance pathway Adipocytokine signaling pathway

-4 -2 0 2 4

Enrichment log(combined score)

-4 -2 0 2 4

Enrichment log(combined score)

Lysine degradation Adherens junction Axon guidance

Ubiquitin mediated proteolysis Ribosome biogenesis in eukaryotes Amyotrophic lateral sclerosis

Alzheimer disease Coronavirus disease

Non-alcoholic fatty liver disease Diabetic cardiomyopathy Huntington disease Prion disease

Parkinson disease Oxidative phosphorylation

Ribosome

-4 -2 0 2 4

Enrichment log(combined score)

-4 -2 0 2 4

Enrichment log(combined score)

Type II diabetes mellitus FoxO signaling pathway

Insulin secretion Renin secretion

Ubiquitin mediated proteolysis

Adherens junction Lysine degradation

Growth hormone synthesis secretion and action

Long-term potentiation cAMP signaling pathway Huntington disease Thermogenesis

Prion disease Diabetic cardiomyopathy

Non-alcoholic fatty liver disease Oxidative phosphorylation Parkinson disease

Ferroptosis Coronavirus disease

Ribosome

-4 -2 0 2 4

Enrichment log(combined score)

**Supplementary Fig. 8**

**
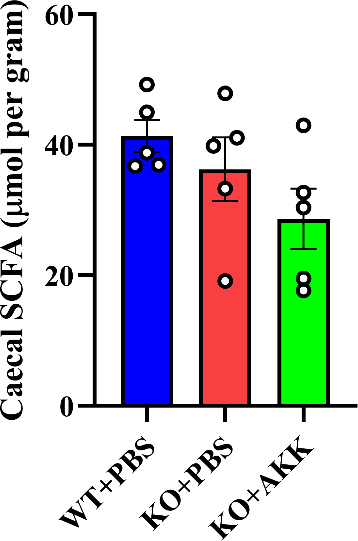

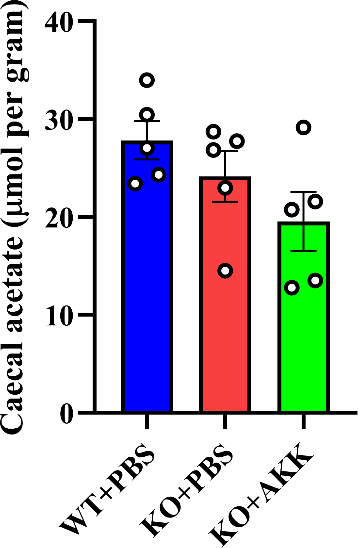
****A B**

**
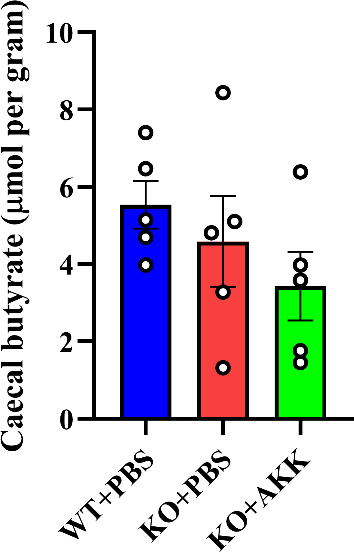

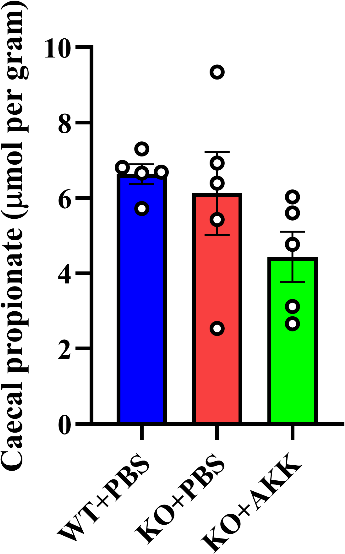
C D**

**
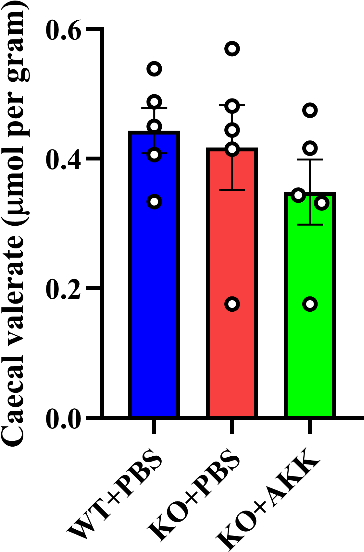

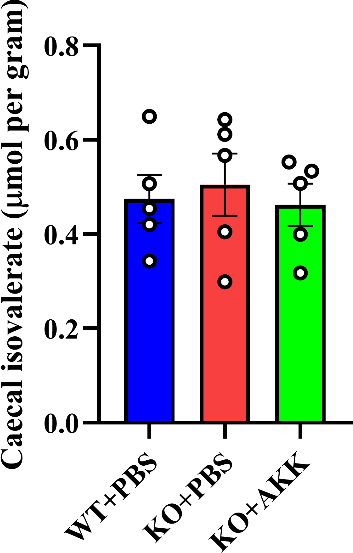

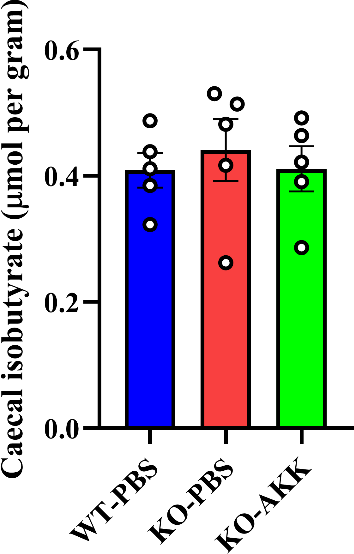
E F G**

**Supplementary Fig. 9**

# A B

### WT+PBS WT+AKK KO+PBS KO+AKK

8


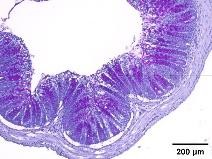

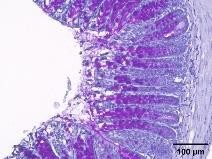

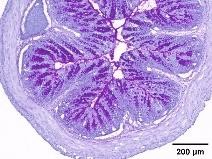

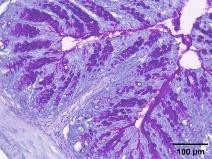

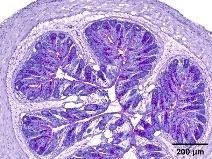

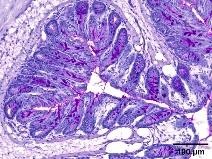

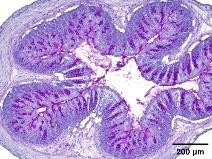

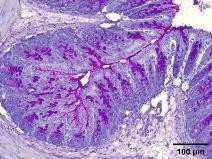

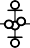

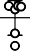


6

Colonic mucus thickness (㎛)

10x

4

2

20x

# C

*Muc2 Zo1 Occludin*

0

###### PBS AKK PBS AKK

### WT KO

2.5 2.5 5


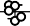

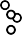

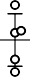

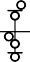


2.0 2.0 4

Relative mRNA expression

Relative mRNA expression

Relative mRNA expression

1.5 1.5 3

1.0 1.0 2

0.5 0.5 1

0.0

PBS AKK PBS AKK

### WT KO

0.0

PBS AKK PBS AKK

### WT KO

0

PBS AKK PBS AKK

WT KO

**Supplementary Fig. 10**

# E

**Negr1 KO mouse**


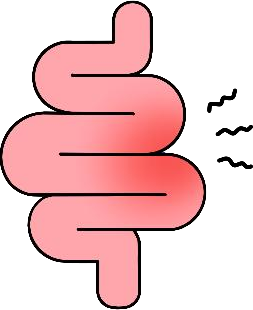

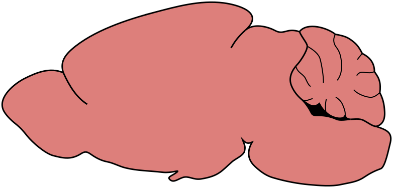

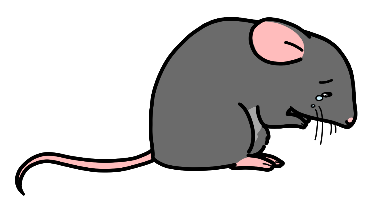

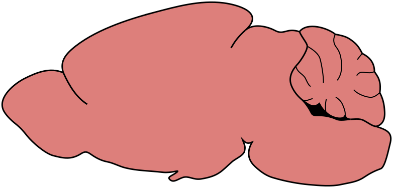

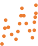

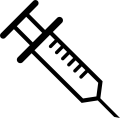

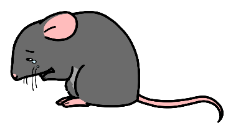

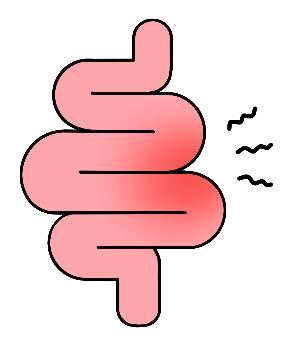

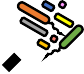

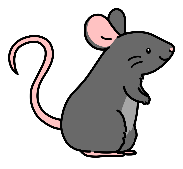


**(Anxiety & Depression)**

- ***Akkermansia* spp. ↓**
- **Interferon-inducible genes ↑**
- ***α*-defensin genes ↓**

## Neuronal atrophy

- **mtDNA-encoded genes ↑**

**Wild-type *Negr1* KO mice**

***Akkermansia* administration**

**Anxiety & depression↓**

- **T cell activation genes ↑**
- **Immune homeostasis genes ↑**

## Reduced Neuronal atrophy

(Dendrite length ↑, Spine density ↑)

## mtDNA-encoded genes ↓

**Supplementary Fig. 11**
